# An automated and combinative method for the predictive ranking of candidate effector proteins of fungal plant pathogens

**DOI:** 10.1038/s41598-021-99363-0

**Published:** 2021-10-05

**Authors:** Darcy A. B. Jones, Lina Rozano, Johannes W. Debler, Ricardo L. Mancera, Paula M. Moolhuijzen, James K. Hane

**Affiliations:** 1 Centre for Crop and Disease Management, School of Molecular and Life Sciences, Curtin University, Perth, Australia; 2 Curtin Medical School, Curtin University, Perth, Australia; 3 Curtin Health Innovation Research Institute, Curtin University, Perth, Australia; 4 Curtin Institute for Computation, Curtin University, Perth, Australia

**Keywords:** Computational biology and bioinformatics, Machine learning, Fungi, Pathogens, Effectors in plant pathology

## Abstract

Fungal plant-pathogens promote infection of their hosts through the release of ‘effectors’—a broad class of cytotoxic or virulence-promoting molecules. Effectors may be recognised by resistance or sensitivity receptors in the host, which can determine disease outcomes. Accurate prediction of effectors remains a major challenge in plant pathology, but if achieved will facilitate rapid improvements to host disease resistance. This study presents a novel tool and pipeline for the ranking of predicted effector candidates—Predector—which interfaces with multiple software tools and methods, aggregates disparate features that are relevant to fungal effector proteins, and applies a pairwise learning to rank approach. Predector outperformed a typical combination of secretion and effector prediction methods in terms of ranking performance when applied to a curated set of confirmed effectors derived from multiple species. We present Predector (https://github.com/ccdmb/predector) as a useful tool for the ranking of predicted effector candidates, which also aggregates and reports additional supporting information relevant to effector and secretome prediction in a simple, efficient, and reproducible manner.

## Introduction

‘Effectors’ are a broad class of cytotoxic, virulence-promoting, or resistance eliciting molecules that are released from pathogen cells to facilitate disease progression. Fungal effectors are a core research area toward improved host disease resistance; however, because they generally lack common features or obvious sequence similarity, discovery of effectors is non-trivial^1–3^. Secreted effector proteins of plant pathogens have been studied more comprehensively in the Oomycetes (a separate lineage of filamentous microbes), in which in silico identification of effectors is more feasible compared to fungi as they exhibit highly conserved sequence motifs (e.g. RXLR, LXLFLAK)^4,5^. In contrast, fungal effectors are highly diverse in sequence and function. This may be a result of their highly plastic genomes, which are diversified by a number of fungal-specific genome mutagenesis mechanisms such as repeat-induced point mutation (RIP)^6,7^ and mesosynteny^8^, as well other genome characteristics common to many pathogens such as the presence of accessory sequences^9^ and lateral gene transfer^10^. Consequently, fungal effector candidate discovery is typically performed using a combination of experimental techniques such as phenotype association and comparative genomics^11–14^, transcriptomics^15–17^, proteomics^18,19^ and GWAS^20,21^. There are, however, some protein characteristics—i.e. structural features, functional domains, signal peptides, amino-acid frequencies—that can be used as an alternative to simple homology searches. Several methods using these characteristics have been developed to identify effector candidates for experimental validation^2^.

In-silico effector prediction has generally targeted small-secreted proteins (SSPs), which typically involves ad hoc, hard set criteria such as a signal peptide, no transmembrane domains outside the signal peptide, small overall size (often < 300AA), and a high number of cysteine amino-acids. These thresholds were based on the properties of early discovered effectors; however, numerous known effectors do not conform to this profile (Supplementary Table S1) and the use of simple hard filters risks excluding these proteins from candidacy. Signal peptide prediction is the most common *in-silico* technique used to refine effector candidates from proteomes^22^, with SignalP the most common prediction tool^23–25^ although other tools are frequently used in combination^26,27^, and different tools can perform better or worse with different protein groups or organisms^22^. Subcellular localisation prediction tools such as TargetP^28^ or DeepLoc^29^ are also frequently used to predict the location of proteins. Their reliability for predicting protein secretion is questionable^22^, but proteins predicted to be localised in organelles might reasonably be excluded. Because most effectors are expected to be free in the extracellular space or host cells, transmembrane domains (TM) are also an important feature for excluding candidates, commonly predicted using TMHMM^30^ or Phobius^26^.

Recently developed machine learning tools tailored to identifying proteins with effector-like properties have presented new opportunities for improving effector prediction pipelines. EffectorP^31,32^ and FunEffector-Pred^33^ use amino acid frequencies, molecular weight, charge, AA k-mers, and other protein characteristics to predict effector-like proteins directly. In combination with secretion prediction, tools like EffectorP and FunEffector-Pred may be a more robust alternative to simple hard filters. LOCALIZER^34^ and ApoplastP^35^, which predict host subcellular or apoplastic localisations, are useful for evaluating candidates but are not necessarily predictive of effector candidature themselves.

While many fungal effectors have previously not had similar sequences in public databases, a small but increasing number of families based on conserved domains or structure are becoming known^2^, including the ToxA-like^36^, MAX^37^, RALPH^38^, and RXLR-like^39^ families. Presence of virulence associated conserved domains (i.e. selected Pfam domains) or effector-like sequences within databases such as the Plant-Host Interactions database (PHI-base)^40^ and the Database of Fungal Virulence Factors (DFVF)^41^, are growing in their relevance. Secondary and tertiary structural modelling and similarity searches against known effectors are not commonly used for high-throughput effector discovery, but this could yet become an important component of future effector prediction pipelines^2^.

Current effector prediction pipelines face two major challenges: (1) the necessity of reducing 10–20 thousand proteins per genome down to a small set of effector candidates that is both reliable and within a number that is feasible for experimental validation, and (2) the amalgamation of outputs from a large and diverse range of bioinformatics tools and methods, for both prediction and informative purposes. Fungal genome datasets typically contain thousands of predicted secreted proteins, of which hundreds of SSPs may be predicted by standard methods^2^. Further filtering or ranking based on supporting data from GWAS, RNAseq, positive selection, or comparative genomics can still generate hundreds of candidates^42–45^. The prioritisation of effector candidates based on simple biochemical properties is, therefore, still relevant to effector prediction. However, there is little consensus on how to combine multiple analyses^22^, and the common use of multiple successive hard filters risks increasing the error with each step, potentially causing good candidates to be excluded. Furthermore, while hard filters are useful for identifying sets of well-defined classes of effectors (e.g. small cysteine rich), these methods do not provide a clear means of prioritising candidates for experimental follow-up.

Saunders et al.^46^ approached this problem by ranking clusters of homologous proteins using multiple e-value like scores based on the expected frequencies of effector-indicating properties of interest within a cluster, and used hierarchical clustering to combine information from the e-value scores and identify extended groups of effector candidates with common features. While this method addresses some of the issues described above, the use of criteria highly specific to that study, and dependency on protein homology clustering potentially limits the broader applicability of this method.

Rank-based methods have been used as a far simpler way of avoiding the exclusion of candidates that lack clearly discriminative features. In this approach, weighted scores are assigned to multiple features that are presumed to be important in determining effector-likelihood, and these weights are summed into a single score that is used to rank candidates^45^. However, these simple combinations of manually assigned feature weights may still fail to place candidate proteins with uncommon characteristics near the top of the list. More sophisticated ranking decisions may come from a group of machine learning techniques called “learn to rank”. Rather than offering a binary classification (i.e. effector or non-effector), these methods attempt to order elements optimally so that more relevant elements are nearer the beginning of the list. Although these algorithms are most often employed in search engine and e-commerce websites, they have been used successfully to combine diverse sources of information and rank protein structure predictions^47^, remote homology predictions^48^, gene ontology term assignments^49^, and predicting protein-phenotype associations in human disease^50^.

In this study, we present a novel tool and pipeline for the ranking of predicted effector candidates—Predector—which interfaces with multiple software tools and methods, aggregates disparate features that are relevant to fungal effector proteins, and ranks effector candidate proteins using a pairwise learning to rank approach. Predector simplifies effector prediction workflows by providing simplified software dependency installation, a standardised pipeline that can be run efficiently on both commodity hardware and supercomputers, and user friendly tabular formatted results. In this study, we compare the ranking performance of Predector against a typical effector prediction method (i.e. signal peptide prediction, transmembrane domain prediction, and EffectorP), on a curated set of confirmed effectors derived from multiple species. While the small number of currently known effectors and relatively loose definition of the group precludes the possibility of perfectly precise effector prediction tools, we present Predector as a tool enabling useful effector candidate ranking alongside supporting information for effector and secretome prediction in a simple, efficient, and reproducible manner.

## Results

**Figure 1:**
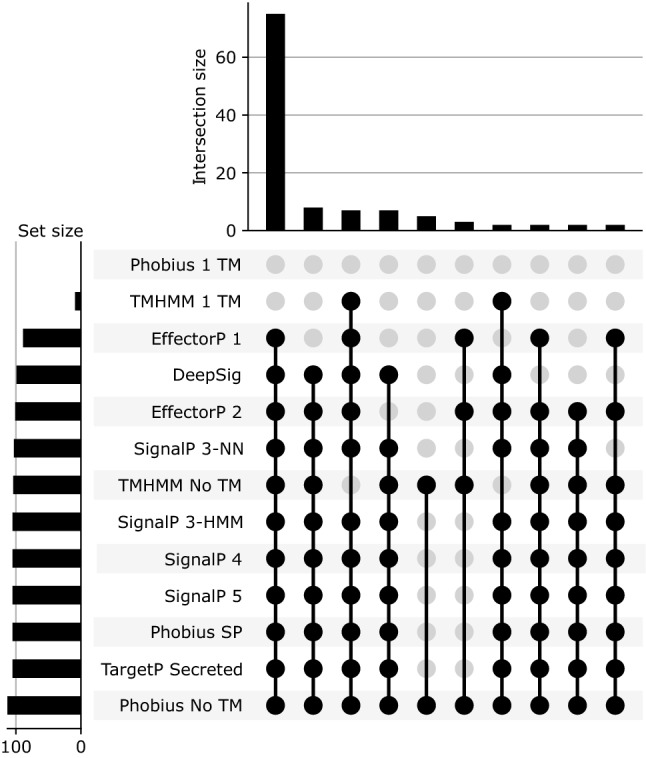
UpSet plot showing predictions of signal peptides, transmembrane domains, and effector-like properties for all known effectors in the training dataset (N = 125). Rows indicate sets of proteins predicted to have a property related to effector prediction (e.g. a signal peptide), with the horizontal bar chart indicating set size. Columns indicate where the horizontal sets intersect with each other, where the vertical bar-chart indicates the number of proteins in that intersection. For clarity, intersections with only 1 member have been excluded, the full plot is presented in Supplementary Data S1:1.

To develop and evaluate the Predector pipeline, a dataset of unprocessed fungal proteins was collected and split into train and test datasets (Supplementary Table S2). The division of protein sequences into training and test datasets was selected to ensure comparability with EffectorP2 and proteins were also clustered to remove highly similar sequences. The datasets included redundancy reduced proteins of known fungal effectors (train: 125, test: 28), fungal proteins in the SwissProt database annotated as secreted (train: 256, test: 64) and non-secreted (train: 8676, test: 2169), and the whole proteomes from 10 well studied fungal genomes (train: 52,224, test: 13,056; Supplementary Table S2). Homologues of known effectors that were not clustered with the other known effectors during redundancy reduction were retained as an informal validation dataset during training and model development. Homologues were not included in training or test datasets during model training or any formal evaluation of Predector score performance, but provide useful supporting information (Supplementary Table S3). The Predector pipeline runs numerous tools related to effector and secretome prediction (Table 3). Benchmarking those tools against the set of confirmed effector proteins in the train dataset, it was observed that the secretion prediction tools were frequently correct with a small number of exceptions (Fig. 1). Signal peptide prediction recall in the training dataset of known effectors ranged from 84% (DeepSig) to 92% (TargetP 2). SignalP 3, 4, 5, and Phobius generally predicted about 90% of effectors to have signal peptides (Fig. 1). Transmembrane (TM) predictors were, as expected, generally not able to predict TM domains in confirmed effectors, with the few single TM predictions by TMHMM or Phobius likely to be mis-predictions within N-terminal signal peptides. In the case of TMHMM, all effectors with at least one TM domain had more than ten AAs predicted to be TM associated in the first 60 residues by TMHMM (Supplementary Data S1:39). Effector prediction tools (EffectorP 1 and 2) were also able to predict most, but not all, of the confirmed effector set. EffectorP correctly predicted 85.6% and 76.8% of effectors in the training dataset for versions 1 and 2, respectively. Evaluation of protein features that might allow for distinction between the different protein classes considered in this study (effectors, effector homologues, secreted proteins, non-secreted proteins, and unlabelled proteomes) identified 12 features that could be used effectively. These included: the proportion of cysteines, small, non-polar, charged, acidic, and basic amino acids; ApoplastP prediction; DeepLoc extracellular or membrane predicted localisations; molecular weight; EffectorP scores, and signal peptide raw scores (see Supplementary Data S1:3–40). These protein properties identified in this study are corroborated by similar findings in the EffectorP studies^31,32^.

To incorporate information from the selected features related to effector and secretion prediction, a pairwise learning to rank model was trained using XGBoost^51,52^. The mean cross validated normalised discount cumulative gain (NDCG)^53^ in the top 500 ranked predictions (NDCG@500) for the hyper-parameter optimised model was 0.93 with standard deviation 0.009, indicating high performance and little effect of substructure within the dataset. The mean NDCG@500 for the train sets within the cross validation was 0.89 (SD 0.02), indicating that the model was not overfitting.

**Figure 2:**
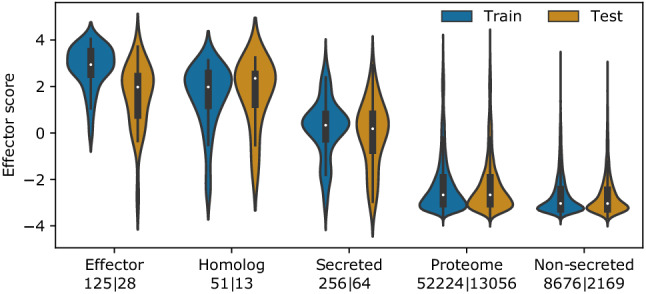
A violin plot showing the distributions of Predector effector ranking scores for each class in the test and training datasets. The effectors consist of experimentally validated fungal effector sequences. “Secreted” and “non-secreted” proteins are manually annotated proteins from the SwissProt database. Proteomes consist of the complete predicted proteomes from 10 well studied fungi (Supplementary Table S2). The number of proteins represented by each violin are indicated on the x-axis.

Benchmarked against a test set (Fig. 2) the Predector model consistently gave higher scores to confirmed effector proteins, and also to homologues of confirmed effectors (on which the model was not trained). Secreted proteins from SwissProt tended to have intermediate scores centred around 0. Non-secreted and the unlabelled proteins were heavily skewed towards more negative scores, with a long tail that included some proteins with high scores (which in the case of proteomes was expected as this dataset may contain novel effectors). The test and train sets showed similar distributions of scores, though there tended to be slightly lower scores for known effectors in the test set.

The main features used by the learning to rank model for sorting effectors from non-effectors in the Predector model were TargetP secretion prediction, SignalP 3-HMM S-scores, SignalP4 D-scores, DeepLoc extracellular and membrane predictions, and EffectorP 1 and 2. TargetP secretion was overwhelmingly the most important feature according to the gain metric (the average increase in predictive score when the feature is used; Supplementary Data S1:43), which was consistent with the observation that it was the most sensitive of the signal peptide prediction methods for effectors (Fig. 1). The most commonly used features were EffectorP 2 pseudo-probabilities, molecular weight, and the proportions of cysteines, basic AAs, non-polar AAs and tiny AAs. Feature importance and boosted trees indicated overall that the Predector model first coarsely sorts proteins into the predicted secretome and non-secreted proteins, then proceeds to separate proteins with effector-like properties from the remainder of the proteins using more decision nodes each with smaller overall gain (Supplementary Data S1:43).

**Figure 3:**
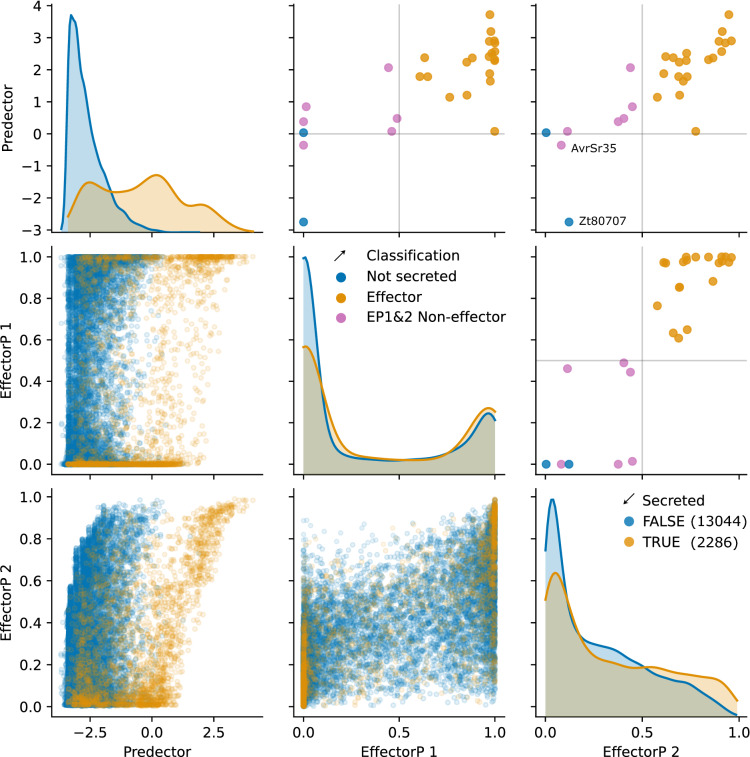
Comparing the scores of Predector with EffectorP versions 1 and 2 for proteins in the testing dataset. Scatter plots in the lower-left corner indicate comparisons of predictive scores between methods, with predicted secreted proteins (any signal peptide and fewer than two TM domains predicted) indicated in yellow, and non-secreted proteins indicated in blue. Density plots along the diagonal indicate distributions of the full test dataset versus predictive scores for each method (indicated along the x-axis), also coloured by secretion prediction as before (Note: there are far more non-secreted than secreted proteins in the dataset). Scatter plots in the top-right corner indicate score comparisons between methods for confirmed effectors, coloured by whether they have been predicted as secreted (criteria as above), or additionally predicted by EffectorP versions 1 or 2. Two proteins that are misclassified by a Predector score > 0 are labelled in the top-right subplot.

Predector separated some proteins predicted to be secreted (i.e. with a signal peptide and fewer than two TM domains), from those that are not (Fig. 3). Most “non-secreted” proteins have a score < 0, while a tri-modal distribution of “secreted proteins” was observed, which spanned the full range of scores and roughly coincided with the distributions of effectors/homologues, SwissProt secreted and the non-secreted/proteome datasets (Fig. 2). This contrasted with EffectorP predictions (which was trained and is intended to be used on secretomes only), which gave poor separation of non-secreted and secreted proteins. EffectorP 1 showed a high bias to predicting proteins as either 0 or 1, indicating that it may be unsuited for ranking and should only be used as a decision classifier with a score threshold of 0.5. EffectorP 2 showed a more continuous separation of known effectors, and was moderately correlated with Predector scores for secreted proteins.

Performance of Predector was compared against a typical effector prediction classifier based on EffectorP 1 and 2, and secretion prediction (defined as any signal peptide prediction and fewer than two predicted TM domains by any method in Table 3). Predector consistently outperformed this combined classifier in terms of classification recall and Matthews correlation coefficient, and in metrics assessing the ranked order of effector candidates using EffectorP scores within the predicted secretomes (Table 1, Supplementary Table S3). While EffectorP was not optimised for effector candidate ranking or intended to be used this way, we note that its probability score could potentially be used for this purpose. We include these comparisons for illustrative purposes rather than as an endorsement of how they should be used. Similarly, although Predector was not intended to be used for effector classification, to offer a fairer comparison with existing classification-based methods we also compared its predictive performance with EffectorP 1 and 2 on the secreted subset, and on the full dataset using the joint estimator of secretion and EffectorP score > 0.5. For the purpose of this comparison, a minimum Predector score of 0 was selected as a classification threshold based on the observation that the model assigns positive scores to effector associated branches in the trees (and negative scores to non-effector associated branches). EffectorP 1 and 2 performed identically in terms of effector classification on our test dataset and gave highly similar results on the training dataset (Supplementary Table S3, Supplementary Data S1:50), though fewer false positives were reported by EffectorP 2. Predector correctly predicted all but two effectors in the full test set, and all but one in the secreted test subset. In contrast, EffectorP 1 and 2 both mis-classified six effectors in the secreted subset, and two known effectors in the test dataset were not predicted to be secreted thus would have been excluded from candidacy by the combined EffectorP-Secretion classifier. It is also worth noting that in this study secretion prediction incorporates multiple methods, whereas many studies rely on a single prediction tool, thus the proportion of potentially missed effector candidates may be higher than we report here. Predector also correctly predicted two confirmed effectors (FolSix12 and BghBEC3) that were not predicted to be secreted as effectors. Although Predector, not being optimised for classification, had a higher false positive rate than EffectorP 1 and 2, it compared favourably for the MCC metric which is considered more reliable for unbalanced datasets^54^.

**Table 1:** Effector prediction and ranking statistics for Predector and a combined classifier based on EffectorP and secretion prediction on the test dataset.

	Full test dataset			Secreted test subset		
	EP1 and Sec	EP2 and Sec	Predector	EP1	EP2	Predector
**Rank**						
Coverage error	–	–	8054	2275	1593	1115
NDCG@50	–	–	0.640	0.615	0.629	0.652
NDCG@500	–	–	0.928	0.916	0.926	0.933
NDCG	–	–	0.447	0.365	0.402	0.448
TP@50	–	–	4	2	2	4
TP@500	–	–	20	13	18	20
**Classification**						
TP	20	20	26	20	20	25
TN	14,450	14,609	14,317	1410	1569	1323
FP	839	680	972	839	680	926
FN	8	8	2	6	6	1
Precision	0.023	0.028	0.026	0.023	0.028	0.026
Recall	0.714	0.714	0.928	0.769	0.769	0.961
FPR	0.055	0.044	0.064	0.373	0.302	0.412
Accuracy	0.944	0.955	0.936	0.628	0.698	0.592
Balanced accuracy	0.829	0.834	0.932	0.698	0.733	0.774
MCC	0.122	0.137	0.149	0.086	0.107	0.118

Test datasets here do not contain any effector homologue sequences. Note that EffectorP is not optimised for ranking tasks and Predector is not optimised for classification. These scores are shown merely for comparison and not necessarily as an endorsement of how they should be used. Coverage error is the index of the last known effector in the test dataset. NDGC is a measure of how often effectors are placed ahead of unlabelled samples in the list sorted by score, penalising incorrect orderings more highly near the top of the list. NDGC@N is the same statistic but only for the top N items in the sorted list. TP, TN, FP, FN are the number of true positives, true negatives, false positives, and false negatives for the classification task, respectively. TP@N indicates the number of known effectors in the top ranked N proteins. Precision indicates how many of the predicted effectors are false positives (unlabelled in this case, so these could be real effectors; higher being better), recall indicates how many of the known effectors are correctly predicted as effectors (higher being better), and FPR (false positive rate) indicated how many of the unlabelled set were incorrectly predicted as effectors (lower being better). Balanced accuracy and MCC are better indicators of model predictive performance than precision for unbalanced data. The secreted test subset consists only of known effector proteins and proteins with a signal peptide (by any method) and fewer than two predicted TM domains (by either TMHMM or Phobius). Correct classification for EffectorP in the full dataset is conditional on secretion prediction by the same criteria as the secreted dataset (SP and < 2 TM). For the same reason, Predector and EffectorP cannot be fairly compared by ranking statistics in the full dataset.

*EP1* effectorP v1, *EP2* EffectorP v2, *Sec* secreted.

To test whether the improved recall observed in the Predector evaluation relative to the EffectorP-Secretion classifier is caused by an overly-relaxed decision threshold, we evaluated the classification performance of each method using a decision threshold that results in the same number of true positives. The score of the 20th highest ranked known effector by Predector had a score of 1.14, and the scores of the 26th highest ranked known effectors by EffectorP 1 and 2 (restricted to the secretome) were 0.114 and 0.0, respectively. We evaluated classification performance of Predector and the combined Secretion/EffectorP2 methods using these new decision thresholds (Supplementary Table S3). Increasing the Predector decision threshold resulted in 411 false positive predictions in the complete test dataset, compared to 680 false positives by the combined Secretion/EffectorP2 at the default decision threshold (0.5, which achieved the same number of true positives). Decreasing the EffectorP2 decision threshold resulted in 1459 false positive secreted proteins, compared to 972 false positive predictions by the Predector default threshold (0, also achieving the same number of true positives). In general, we observed that Predector maintained higher recall with increasing decision thresholds than EffectorP1 and 2 within the predicted secreted test data, and we see higher precision near the larger Predector scores compared to EffectorP1 and 2 (Supplementary Data S1:46,47).

To further evaluate Predector we assessed 35 new effector sequences identified since the pipeline and model was developed, that were distinct from the existing set of confirmed effectors. We identified similar sequences in the existing training dataset by searching new effector sequences against the non-homology reduced training dataset using MMSeqs version 13.45111 (e-value ≤ 1e−5; Supplementary Table S4)^55^. Any new sequences matching sequences that were designated as belonging to the classes “effector” or “homolog” were discarded, leaving only proteins that exclusively matched the “unlabelled” or “non-effector” sequences (i.e. secreted, non-secreted, and proteomes). Of these 35 new effectors, 9 were unlike any other sequences in the existing dataset, 11 were similar to proteins in both the “train” and “test” partitions, 14 were similar only to proteins in the “train” partition, and 1 was similar to proteins in the “test” partition. The Predector scores of these new effector proteins do not appear to be biased by presence of homologues in the training negative dataset, with the highest scoring protein MoHrip2 (Predector score: 2.62) having two highly similar proteins in the *Fusarium* *oxysporum* f. sp. *lycopersici* proteome, both designated as non-effectors in the “train” partition. Twenty-seven of these new proteins had Predector scores greater than 0, and 33 had scores greater than − 0.5, meaning that most would be well separated from the bulk of most proteomes. In contrast, EffectorP 1 and 2 predicted only 15 and 9 of these new proteins as effectors, respectively. Five proteins were missing a signal peptide prediction by at least one method, and 7 proteins had at least 1 transmembrane domain predicted by either Phobius or TMHMM (all but 1, MoCDIP8, were attributable to the signal peptide regions). Two proteins, MoCDIP8 and MoCDIP13, had very low Predector scores (− 2.68 and − 2.75, respectively). MoCDIP8 has two predicted transmembrane domains, no signal peptide predictions, and was not predicted to be an effector by EffectorP 1 or 2. MoCDIP13 was predicted to be an effector by EffectorP 1 but not 2, and did have a signal peptide predicted by SignalP 3 and Phobius, but not by the newer prediction methods (SignalP 4 or 5, TargetP, Deepsig). Additionally, DeepLoc predicted MoCDIP13 to be membrane-associated and mitochondrially-localised.

For a set of 15 fungal proteomes retained separately for evaluation (Table 2, Supplementary Table S2, Supplementary Data S2), the numbers of predicted secreted and/or effector-like proteins were generally higher in pathogenic species than in typically saprotrophic species. Predector predicted on average 6.9% of proteins in pathogens to have a score greater than 0, but only 3.5% in saprotrophs. For pathogens there was an average of 6.2 effector homologues in the 50 highest scoring predictions by Predector, but only 1.5 in saptrotrophs. The combined Secretion/EffectorP classifiers predicted on average 4.6% and 4.1% of pathogen proteins to be effector candidates for EffectorP versions 1 and 2, respectively; but only 2% and 1.7% on average in saprotrophs. Generally, a smaller proportion of the predicted secretomes in saprotrophs had high Predector or EffectorP2 scores compared with the secretomes of pathogens (Supplementary Data S1:51–54, Supplementary Data S2). Predector running with the default configuration processed whole fungal proteome datasets with an average rate of 1814 proteins per hour on a cloud instance with four CPUs (AMD EPYC vcpus, 16 Gb RAM), and 4514 proteins per hour on a partially occupied single HPC node with 16 CPUs (Intel(R) Xeon(R) CPU E5-2680 v4 @ 2.40 GHz, 48 Gb RAM). Predector can be configured to optimise for different environments to improve utilisation of very large compute resources.

**Table 2:** Predector results on pathogen and saprobe proteomes held out of the training set.

Organism	Class^a^	# proteins	# secreted	Predector	EP1 and Sec	EP2 and Sec	#homologs in top 50	#Pfam domain in top 50
*Austropuccinia* *psidii* Au_3	B	35,196	3606 (10%)	1271 (4%)	1272 (4%)	1115 (3%)	2	0
*Blumeria* *graminis* f. sp. *tritici* 96224	B	8347	1612 (19%)	696 (8%)	694 (8%)	540 (6%)	20	0
*Melampsora* *larici-populina* 98AG31	B	16,372	2366 (14%)	1282 (8%)	914 (6%)	924 (6%)	1	0
*Venturia* *inaequalis* ICMP13258/MNH120	B	13,233	2212 (16%)	1326 (10%)	740 (6%)	711 (5%)	1	1
*Leptosphaeria* *maculans* NzT4	H	14,026	2249 (16%)	868 (6%)	750 (5%)	559 (4%)	9	9
*Zymoseptoria* *tritici* 3D1	H	11,991	1705 (14%)	971 (8%)	505 (4%)	475 (4%)	4	1
*Alternaria* *brassicicola* BMP1950	N	10,688	1444 (14%)	707 (7%)	308 (3%)	305 (3%)	6	11
*Pyrenophora* *tritici-repentis* M4	N	13,795	1561 (11%)	850 (6%)	347 (3%)	368 (3%)	6	8
*Fusarium* *oxysporum* f. sp. *melonis* 26406	W	26,719	3323 (12%)	1464 (5%)	763 (3%)	710 (3%)	7	8
*Komagataella* *phaffii* GS115	S	5040	389 (8%)	76 (2%)	67 (1%)	65 (1%)	0	4
*Schizosaccharomyces* *pombe* 972h-	S	5134	349 (7%)	97 (2%)	58 (1%)	44 (1%)	1	3
*Serpula* *lacrymans* S7.9	S	14,495	1507 (10%)	487 (3%)	449 (3%)	297 (2%)	3	9
*Trichoderma* *reesei* QM6a	S	9115	1134 (12%)	529 (6%)	207 (3%)	176 (2%)	2	5
*Uncinocarpus* *reesii* 1704	S	7798	766 (10%)	289 (4%)	161 (2%)	149 (2%)	2	5
*Yarrowia* *lipolytica* CLIB122	S	6448	704 (11%)	257 (4%)	128 (2%)	122 (2%)	1	2

Class indicates the lifestyle of the fungus. Proteins were considered to be secreted if they have a secretion signal predicted by any method and fewer than two predicted transmembrane domains. Predector indicates the number of proteins with a Predector ranking score > 0. EffectorP 1 (EP1) and 2 (EP2) predictions were conditional on secretion and used the default 0.5 decision threshold. The number of protein sequence similarity matches to known effectors and matches to Pfam domains with putative virulence functions are noted for the top 50 candidates by ranked by Predector scores.

^a^Main lifestyle classes of each fungus. *B* Biotroph, *H* Hemibiotroph, *N* Necrotroph, *W* Wilt, *S* Saprotroph.

## Discussion

The Predector pipeline unites, for the first time, numerous computational tasks commonly involved in effector and secretion prediction to determine a ranked set of candidate effectors from unprocessed (immature) proteins, simplifying complex data gathering steps. The effector ranking model run as part of Predector provides additional benefits over the standalone use of its composite tools, in combining their individual strengths while being less prone to their weaknesses. It was observed that while the most recently published effector prediction tool available—EffectorP 2^32^—performed well as a very specific classifier, it still missed several confirmed effectors. The preliminary step of secretion prediction can also be error prone, and the combined false positives from both effector and secretion prediction methods, coupled with their common implementation as hard filters, may result in many genuine candidate effectors being discarded. For this reason, we propose that ranking and clustering methods should be preferred over hard filters for prioritising effector candidates.

In terms of effector candidate ranking, EffectorP 2 performed reasonably well for ordering confirmed effectors based on probability score but was not designed to be used in this way and these ranks are not reliable when applied to non-secreted proteins. Predector maintained higher recall with higher scores (Supplementary Data S1:46, 47, Table 1) and achieved comparable or better precision than EffectorP 2 alone for higher effector scores when restricted to the predicted secreted proteins. Although the recall scores for Predector were very high, Predector also predicted 292 more false positives in the test dataset (N = 15,317) than the commonly used method of combining a predicted secretion hard filter with EffectorP 2 (Table 1). We argue that in the context of effector prediction from whole proteomes, prioritising recall over metrics that emphasise minimising the number of false positives is less likely to exclude genuine novel effectors. We also demonstrated that increasing the decision threshold of Predector can achieve similar recall as EffectorP based classifiers but with 269 fewer false positives. The converse is not true, EffectorP decision thresholds cannot be decreased to increase recall without yielding many more false positives than Predector. This demonstrates how the focus of Predector on ranking rather than classification confers flexibility as a decision support tool and mitigates many of the issues associated with lower precision at a given decision threshold. This is especially true for the top-scoring predictions of Predector and EffectorP, which are more likely to selected for experimental validation and thus potentially more indicative of the relative success of either method than the metrics which consider the evaluation sets as a whole. Our benchmarks demonstrated that Predector ranked twice as many known effectors as EffectorP within their respective 50 top-scoring predictions (Table 1), although the relatively low TP@50 numbers of both methods highlight the considerable remaining challenges in accurately classifying effectors. Moreover, unlike the commonly used secretion/EffectorP pipeline, Predector is also able to give reliable ranks to proteins without predicted signal peptides. Thus, while Predector is not intended to be used as a classifier and users should consider scores as arbitrary numbers used to order candidates, we demonstrate its utility as a highly sensitive method for combined secretion and effector prediction, and suggest a decision threshold (score) of 0 or 1 (depending on user preference for precision or recall) for summarisation purposes alongside standard EffectorP and secretion classifiers (which can be obtained from Predector output). Encouragingly, we observed that Predector was capable of giving positive scores to known effectors which were not predicted to have a signal peptide (e.g. test: FolSix12, train: Vdlsc1, Zt103264, PGTG_10537.2, PGTG_16791, BgtAVRa10, FocSix8, BgtAVRk1) or had multiple predicted transmembrane domains (e.g. test: BghBEC3) and thus would have failed to be predicted by alternate methods with a secretion prediction hard filter. We also observed improved recall over the Secretion/EffectorP combined classifier in a set of novel effectors that were identified after model development.

While Predector and other effector prediction tools are not intended to be applied to non-pathogens, for benchmarking purposes within this study we included several saprotroph species. Saprotrophs were generally predicted to have smaller secretomes than pathogenic species, and fewer effector-like proteins predicted by EffectorP1 and 2, and Predector (using a decision threshold of 0). As observed with the test dataset, Predector generally predicted more effector candidates than either secretion/EffectorP combined classifier across all predicted proteomes, including those of pathogens and saprotrophs. However as we have previously noted the focus of Predector on ranking means that users can expect better predictions with higher ranks (scores), and the distributions of rank scores across saprophyte proteomes differed from pathogens, with the maximum range of scores in saprotrophs being notably lower than those of pathogen proteomes (Supplementary Data S1:51–54). Saprobes are not expected to possess effector proteins that facilitate plant-host infection but may still possess proteins with similar functional or physical properties. Some saprophytic fungi are opportunistic pathogens of plants and animals (e.g. *Aspergillus* *flavus*^56^ and *Neurospora* *crassa*^57^), so some overlap with protein functions relevant to plant host interactions is possible. For example, both plant pathogens and saprobes secrete large suites of carbohydrate-active enzymes (CAZymes) and other proteins with degrading or scavenging functions, which often possess similar basic characteristics as effector proteins (e.g. secreted, small, cysteine rich, charged) and in some cases are genuine effectors (e.g. LysM effectors or *Fusarium* *graminearum* XYLA). Saprobes are also in competition with other organisms and secrete antagonists of these potential competitors, such as the yeast killer toxins^58,59^, which may possess properties similar to known plant-pathogen effector proteins. Evaluation of our saprotroph benchmarks must be considered in light of current progress in fungal plant-pathogen effector prediction. While overall trends show Predector and other tools report fewer highly-ranked predictions in saprotrophs than pathogens, with current methods being predominantly based on generic protein properties, it is inevitable that mis-predictions will be made across an entire proteome dataset. We suggest that this further highlights the importance of post-predictive ranking, and in this case comparison of relative rank distributions between pathogen and non-pathogen species.

The predictive rankings provided by Predector are complemented with additional information that can be used to manually evaluate groups of effector candidates, and represents a comprehensive summary of various predicted types of proteins within a fungal proteome dataset, including candidate pathogenicity effectors, effector homologues, predicted secreted proteins, and carbohydrate-active enzymes (CAZymes)^60^. Predector reports the results of database searches against PHI-base, a curated set of known fungal effectors, Pfam domains, and dbCAN HMMs. We recommend that users examine the functionally annotated candidates closely, particularly with respect to homologues of confirmed effectors, prior to consideration of candidates ranked by Predector scores. Similarly, supplementation with experimental evidence or information derived from external tools and pipelines will further improve the utility of the Predector outputs, e.g. selection profiles derived from pan-genome comparisons^45,61^, presence-absence profiles in comparative genomics, genome wide association studies, differential gene expression, or pathogenicity-relevant information relating to the genomic landscape: the distance to DNA repeats, telomeres or distal regions of assembled sequences^9,62^; or codon adaptation. By selecting indicators of general effector properties or molecular interactions of interest, and sorting these lists first by those functionally-guided features and then by Predector score(s), users gain a rich and clear guide for prioritising candidates before proceeding to more resource-expensive experiments (e.g. cloning or structure modelling).

Among known effectors there is considerable diversity of their molecular roles and functions. The modern plant pathology community has yet to come to firm agreement on the broad definition of an effector beyond the classical large-effect genetic models that clearly determine host-specificity, or to refine a broader definition with effector sub-types. Effectors may promote virulence through directly targeting and disrupting host cell biological processes, including ribogenesis, photosynthesis or mitochondrial activity. In contrast various extracellular chitin-binding proteins have also long been described as effectors, yet promote virulence through passively protecting the pathogen cell from host PAMP and DAMP recognition. CAZymes are not typically considered to act as effectors, yet there are several examples of secreted CAZymes that are reported as virulence factors or may be recognised by host major resistance genes^40^. Furthermore, the focus of many effector prediction methods (including Predector) on biochemical or functional aspects of effector proteins also neglects the crucial contribution of host R- and S-proteins in molecular pathogen-host interactions, which must be determined experimentally. An inclusive predictive model spanning diverse effector types may not offer a reliable pathway to rapid effector identification, rather they are likely to focus on general biochemical properties unrelated to necrotrophic, virulence, or avirulence activities, e.g. that would enable the majority to interact with membranes and translocate into a host cell or to function in the apoplast. We present Predector as a reasonable compromise between functional diversity and common purpose, accounting for this inherent diversity through incorporation of multiple predictive methods. Additionally, with rapidly decreasing costs of genome sequencing and improvements to the automation of genome analysis and gene feature annotation, the availability and utility of fungal pathogen genomes is steadily increasing^63^. There is a growing need for tools which will minimise the effects of poor data quality control and ensure reproducibility and comparability across multiple genome resources. The Predector pipeline is an important time-saving tool which applies a standardised and reproducible set of tests method for compiling supporting information relevant to fungal effectors, and for the ranking of predicted effector candidates.

## Methods

### Pipeline implementation

The Predector pipeline runs a range of commonly used effector and secretome prediction bioinformatics tools for complete predicted proteome, accepted as input in FASTA formatted files (Table 3), and combines all raw and summarised outputs into newline-delimited JSON, tab-delimited text and GFF3 formats. The pipeline is implemented in Nextflow (version > 20)^64^, and a conda environment and Docker container are available for easy installation of dependencies, with scripts to integrate user-downloaded proprietary software into these environments. Predector is available from https://github.com/ccdmb/predector.

**Table 3:** Bioinformatics tools and methods integrated into the Predector pipeline.

Software	Description	References
**(A) Localisation**		
SignalP v3.0, 4.1g, 5.0b	Extracellular secretion via signal peptide. Both NN and HMM methods are run for v3.0. Eukaryotic types specified	^23–25^
Deepsig commit 69e01cb	Extracellular secretion. *-k euk	^27^
Phobius 1.01	Extracellular secretion	^26^
LOCALIZER v1.0.4	Host sub-cellular localisation. Using predicted mature proteins from SignalP 5.0b. *-e -M	^34^
ApoplastP v1.0.1	Apoplast-specific localisation	^35^
DeepLoc v1.0	Sub-cellular localisation	^29^
TargetP v2.0	Sub-cellular localisation. *-org non-pl	^28^
TMHMM v2.0c	Membrane localisation via transmembrane domains. *-d	^30^
**(B) Effector-like properties**		
EffectorP v1.0, 2.0	Probabilistic prediction of effector likelihood	^31,32^
EMBOSS: pepstats v6.5.7	Amino acid properties and frequencies	^65^
**(C) Functional annotation**		
HMMER (vs dbCAN v8) v3.2.1	Used to search dbcan	^60,66^
MMSeqs2 v10-6d92c (vs PHIBase v4.9)	Used to search phibase. *--max-seqs 300 -e 0.01 -s 7 --num-iterations 3 -a	^40,55^
MMSeqs2 v10-6d92c (vs known effectors in Supplementary Table S2)	*--max-seqs 300 -e 0.01 -s 7 --num-iterations 3 -a	^55^
PfamScan (vs Pfam v33.1)	With active site prediction. *-as	^67^

*Non-default parameters are indicated where applicable.

### Datasets

The training and evaluation datasets consisted of confirmed fungal effectors, fungal proteins with confirmed subcellular localisation, and an ‘unlabelled’ fungal protein set derived from whole proteomes of well-annotated, model fungal species. The experimentally confirmed effector protein dataset was curated from literature, PHI-base^40^, and EffectorP^31,32^ training datasets (Supplementary Table S2). Effector homologues were also identified from literature (Supplementary Table S2) and by searching the UniRef-90 fungal proteins (UniProtKB query: taxonomy:"Fungi [4751]" AND identity:0.9, UniProt version 2020_01, Downloaded 2020-06-01) using MMSeqs2 version 11-e1a1c^55^ requiring a minimum reciprocal coverage of 70% and a maximum e-value of 10^–5^ (-e 0.00001 --start-sens 3 -s 7.0 --sens-steps 3 --cov-mode 0 -c 0.7). Fungal proteins with experimentally annotated sub-cellular localisation were downloaded from UniProtKB/SwissProt (version 2020_01, downloaded 2020-06-01), and were labelled “secreted” (non-transmembrane) or “non-secreted” (membrane associated, endoplasmic reticulum localised, golgi localised, and Glycosylphosphatidylinositol (GPI) anchored). UniProtKB download queries are provided in Supplementary Table S2. The ‘unlabelled’ whole proteome dataset was derived from well-studied pathogens and non-pathogens, with at least one representative chosen from a range of trophic phenotypes^68^: monomertrophs/biotrophs: *Austropuccinia* *psidii*^69^, *Blumeria* *graminis* f. sp. *hordei*^70^, *Blumeria* *graminis* f. sp. *tritici*^71^, *Melampsora* *lini*^72^, *Melampsora* *larici-populina*^73^, *Puccinia* *graminis* f. sp. *tritici*^74^, *Venturia* *inaequalis*^75^; polymertrophs/necrotrophs—*Alternaria* *brassicicola*^7^, *Parastagonospora* *nodorum*^45^*,* *Pyrenophora* *tritici-repentis*^76^, *Pyrenophora* *teres* f. *teres*^77^, and *Pyrenophora* *teres* f. *maculata*^77^; mesotrophs/hemibiotrophs—*Leptosphaeria* *maculans*^43^, *Zymoseptoria* *tritici*^78,79^, *Passalora* *fulva*^80^; wilts/vasculartrophs—*Fusarium* *oxysporum* f. sp. *lycopersici*^81,82^, *Fusarium* *oxysporum* f. sp. *melonis*^83^; and saprotrophs (or opportunistic monomertroph/biotroph), *Komagataella* *phaffii* (aka *Pichia* *pastoris*)^84^, *Neurospora* *crassa*^85^, *Schizosaccharomyces* *pombe*^86^, *Serpula* *lacrymans*^87^, *Trichoderma* *reesei*^88^, *Uncinocarpus* *reesii*^89^, *Yarrowia* *lipolytica*^90^ (Supplementary Table S2). Fifteen of the 25 proteomes above were retained as a separate dataset for final evaluation (Supplementary Table S2). The remainder of the datasets were combined, and redundant sequences were removed to prevent the undue influence of conserved or well-studied sequences with multiple records. Redundancy was reduced by clustering proteins with MMSeqs2 version 11-e1a1c^55^ requiring a minimum reciprocal coverage of 70% and minimum sequence identity of 30% (--min-seq-id 0.3 --cov-mode 0 -c 0.7 --cluster-mode 0). A single sequence was chosen to represent a set of clustered, redundant sequences, which was prioritised based on supporting information (in order of preference): known effector, SwissProt secreted, SwissProt non-secreted, proteome/effector homologue, longest member of cluster. Clusters that corresponded to the known effectors from the EffectorP 2^32^ training and test data sets were automatically assigned to training and test data sets in this study. A randomly selected subset of 20% of the remaining representative members of clusters were also assigned to the test dataset. Clusters corresponding to effector homologues were placed in the training and testing datasets alongside their homologous known effectors, and were used as an informal validation dataset during model development, so did not contribute directly to the model itself nor any performance metrics. Data and scripts used for generating the datasets are available at https://doi.org/10.5281/zenodo.5225297.

### Learning to rank model training

A “learning to rank” pairwise machine learning method based on LambdaMart^52^ was developed using XGBoost^51^ to prioritise effectors. Effector homologues in the training data set were held out as an informal validation set, known effector proteins were considered relevant (priority 2), and all other proteins in the train dataset were considered irrelevant (priority 1). To mitigate issues caused by unbalanced class sizes, training data were weighted for effectors as #irrelevant/#relevant and unlabelled proteins were given weight #relevant/#irrelevant. A subset of features output by the Predector pipeline and model constraints for the direction of effect (indicated in brackets as + or − when a constraint was applied; + indicating that increasing values of the feature can only contribute positively towards effector prediction) were selected based on the distributions of parameters in Supplementary Data S1:3–40: molecular weight, proportion of cysteines, proportion of tiny AAs (Gly, Ala, Ser and Pro), proportion of small AAs (Thr, Asp and Asn), proportion of non-polar AAs, proportion of basic AAs, EffectorP 1 probability (+), EffectorP 2 probability (+), ApoplastP probability (+), TMHMM TM count (−), TMHMM expected TM residues in first 60 AAs, Phobius TM count (−), DeepLoc membrane probability (−), DeepLoc extracellular probability (+), DeepSig signal peptide (SP) prediction (+), Phobius SP prediction (+), SignalP 3 neural network D-score (+), SignalP 3 HMM S-score (+), SignalP 4 D-score (+), SignalP 5 SP probability (+), and TargetP secreted probability (+). The hyperparameters max_depth, min_child_weight, gamma, lambda (L2 regularisation), subsample (dropout), colsample_bytree, eta (learning rate), and num_boost_round (number of boosted trees) were optimised by maximising the normalised discounted cumulative gain (NDCG)^53^ for the highest 500 ranked proteins (NDCG@500) in fivefold cross validated training. The final model was trained using the optimised hyper-parameters.

### Model and score evaluation

The learning to rank model and EffectorP pseudo-probabilities were evaluated using rank summarisation statistics using the scikit-learn library^91^, which included the coverage error (the rank of the lowest scoring effector), label ranking average precision (LRAP; average proportion of correctly labelled samples with a lower score than each position in the sorted results), the label ranking loss (the average number of results that are incorrectly ordered), and the normalised discount cumulative gain (NDCG; the sum of all ranking priorities divided by the log_2_ of the rank position in the sorted list (DCG), normalised by the best theoretically possible DCG score)^53^. NDCG, LRAP, and label ranking loss were also evaluated for the top 50, 500, and 5000 proteins (indicated with the suffix @50, @500, or @5000). The number of true positives within the top 10, 50, 100, and 500 ranked candidates were also computed. Additionally, to compare classification performance of the learn to rank model with the combined EffectorP and secretion prediction decisions, a decision threshold of 0 was set for the learn to rank model (with > 0 indicating an effector prediction), and the classification metrics precision (the proportion of predicted effectors that are labelled as true effectors), recall (the proportion of known effectors that are predicted to be effectors), accuracy (the fraction of correct predictions), balanced accuracy (the arithmetic mean of precision and recall for binary cases like this, and is less affected by unbalanced data-sets than accuracy), F1 score (the harmonic mean of precision and recall), and Matthews correlation coefficient (MCC). For unbalanced datasets like the training set of effectors and non-effectors, MCC is considered a more reliable indicator of classification model performance than the other methods mentioned above^54^. Additionally, to evaluate the performance at different decision thresholds, the precision, recall, and MCC were calculated for 100 score thresholds along the range of each score, and the receiver operating characteristic (ROC) curves were plotted.

For the effector ranking scores, only known (i.e. experimentally validated) effectors were used as the relevant (positive) set with the irrelevant (negative or unlabelled) set consisting of secreted, non-secreted, and proteomes. Because EffectorP is intended to be run on secreted datasets, ranking statistics were only calculated for the subset of proteins that were predicted to have a signal peptide (by any method) and with fewer than two predicted TM domains (by either Phobius or TMHMM), and classification statistics were considered on both this secreted subset, and as a combined classifier (secretion and EffectorP prediction) on the whole datasets.

## Supplementary Information

Supplementary Information 1.

Supplementary Information 2.

Supplementary Information 3.

Supplementary Information 4.

Supplementary Information 5.

Supplementary Information 6.

## Data Availability

All Predector results, and sources of data are provided in the supplementary materials. Sequences used for training and evaluation in this study are available online at: https://doi.org/10.5281/zenodo.5225297, under the “processed” folder. The Predector pipeline is available online at: https://github.com/ccdmb/predector (git tag: 1.0.0). The learning to rank model and utility scripts used as part of the pipeline are available online at: https://github.com/ccdmb/predector-utils (git tag: 0.2.0).
