# Publisher Correction: An automated and combinative method for the predictive ranking of candidate effector proteins of fungal plant pathogens

**DOI:** 10.1038/s41598-021-03673-2

**Published:** 2021-12-13

**Authors:** Darcy A. B. Jones, Lina Rozano, Johannes W. Debler, Ricardo L. Mancera, Paula M. Moolhuijzen, James K. Hane

**Affiliations:** 1 Centre for Crop and Disease Management, School of Molecular and Life Sciences, Curtin University, Perth, Australia; 2 Curtin Medical School, Curtin University, Perth, Australia; 3 Curtin Health Innovation Research Institute, Curtin University, Perth, Australia; 4 Curtin Institute for Computation, Curtin University, Perth, Australia

Correction to: *Scientific Reports* 10.1038/s41598-021-99363-0, published online 05 October 2021

The original version of this Article contained an error in Table 2, where the ‘# secreted’ was incorrectly stated for the organism ‘*Austropuccinia* *psidii* Au_3’. The correct and incorrect values appear below.

Incorrect:

**Table:** 

Organism	# secreted
*Austropuccinia* *psidii* Au_3	1

Correct:

**Table:** 

Organism	# secreted
*Austropuccinia* *psidii* Au_3	3606 (10%)

The original Article has been corrected.

