# Supplementary material for: An automated and combinative method for the predictive ranking of candidate effector proteins of fungal plant pathogens
Source: Sci Rep. 2021 Oct 5;11:19731. doi: 10.1038/s41598-021-99363-0 (PMC8492765; doi:10.1038/s41598-021-99363-0)
Supplement: Supplementary file 1 — Supplementary Information 1. [file 41598_2021_99363_MOESM1_ESM.docx]

**Supplementary Data S1.** Supporting figures. Captions included inside document.


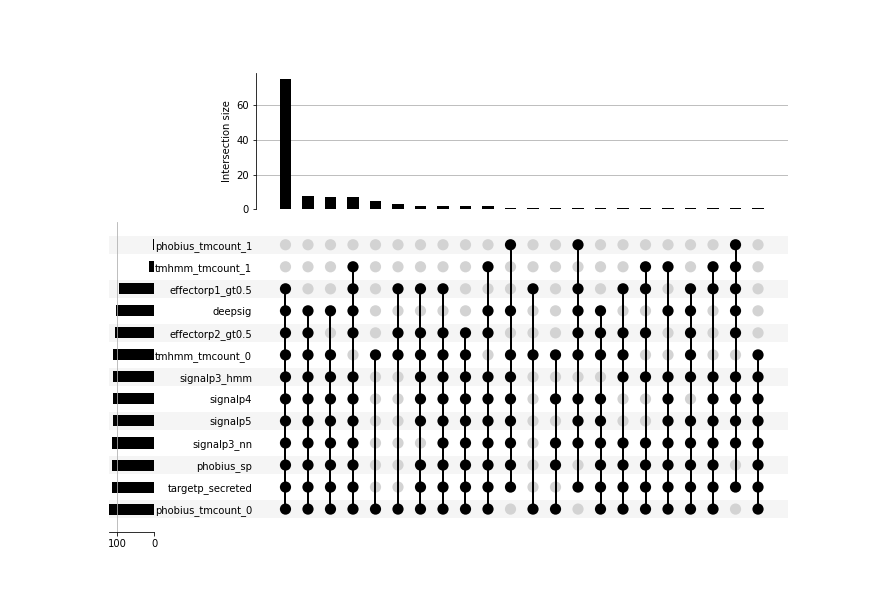


Supp. Figure 1.

UpSet plot showing predictions of signal peptides, transmembrane domains, and effector-like properties for all known effectors in the training dataset (N=125). Rows indicate sets of proteins predicted to have a property related to effector prediction (e.g. a signal peptide), with the horizontal bar chart indicating set size. Columns indicate where the horizontal sets intersect with each other, where the vertical bar-chart indicates the number of proteins in that intersection. This is the same as figure 1, but with all intersections included.


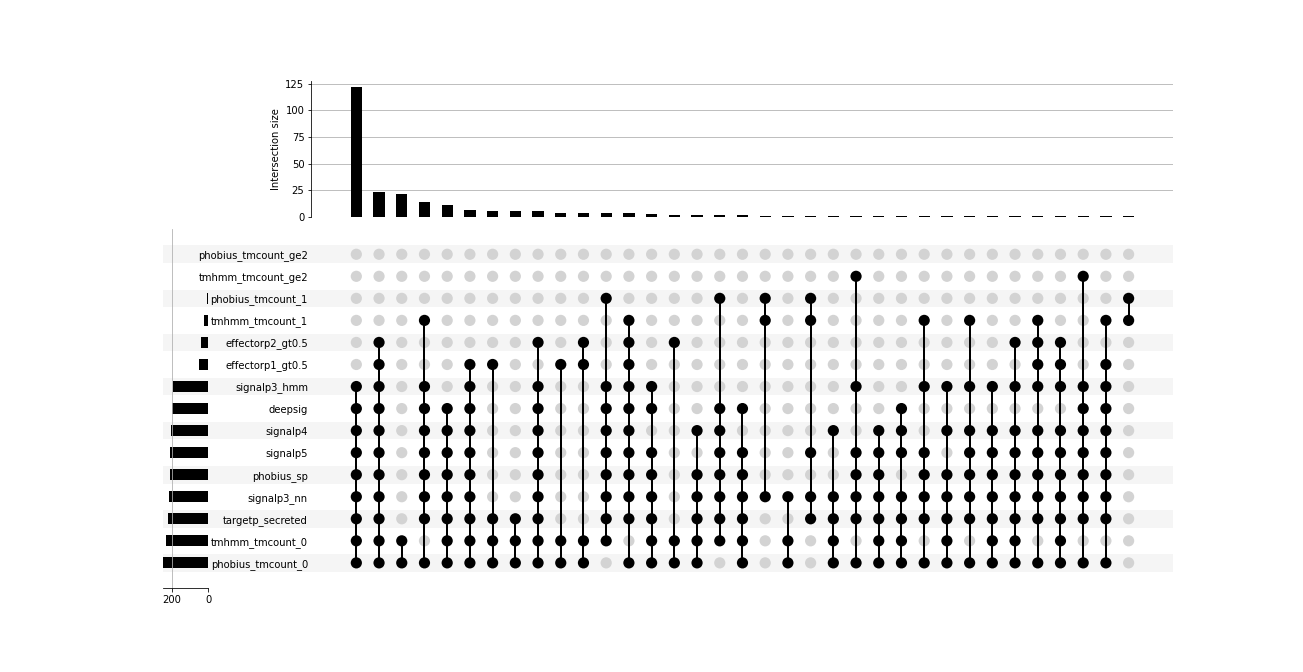


Supp. Figure 2.

UpSet plot showing predictions of signal peptides, transmembrane domains, and effector-like properties for all fungal proteins in SwissProt annotated as secreted in the training dataset (N=256). Rows indicate sets of proteins predicted to have a property related to effector prediction (e.g. a signal peptide), with the horizontal bar chart indicating set size. Columns indicate where the horizontal sets intersect with each other, where the vertical bar-chart indicates the number of proteins in that intersection.


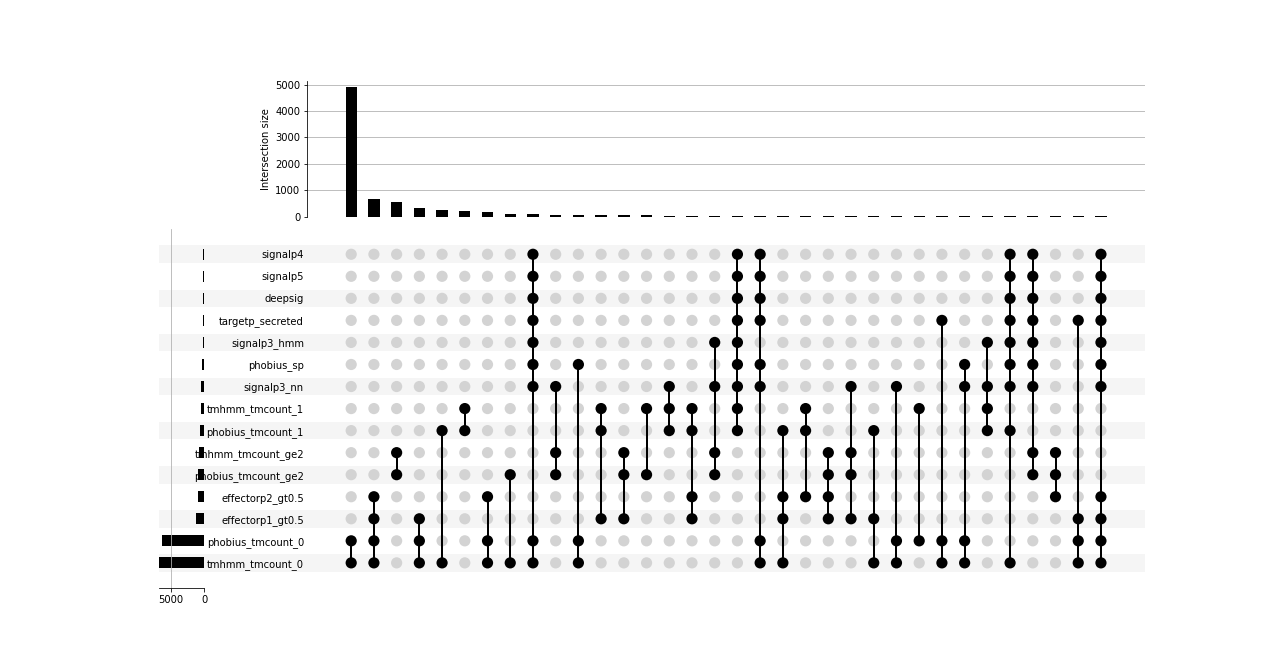


Supp. Figure 3

UpSet plot showing predictions of signal peptides, transmembrane domains, and effector-like properties for all fungal proteins in SwissProt annotated as non-secreted in the training dataset (N=8676). Rows indicate sets of proteins predicted to have a property related to effector prediction (e.g. a signal peptide), with the horizontal bar chart indicating set size. Columns indicate where the horizontal sets intersect with each other, where the vertical bar-chart indicates the number of proteins in that intersection. Intersections with less than 10 members are removed for clarity.


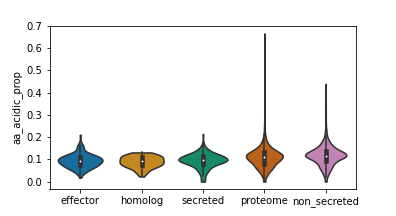


Supp. Figure 4

Proportion of acidic (B, D, E or Z) amino acids in proteins in training datasets.


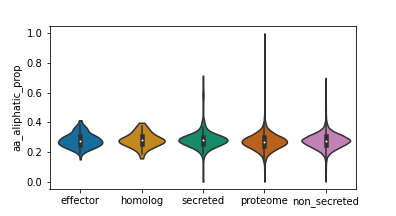


Supp. Figure 5

Proportion of aliphatic (A, I, L, or V) amino acids in proteins in training datasets.


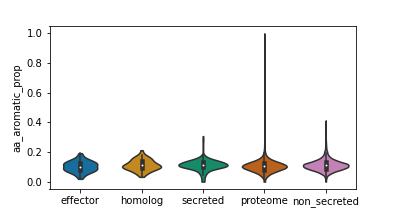


Supp. Figure 6

Proportion of aromatic (F, H, W, or Y) amino acids in proteins in training datasets.


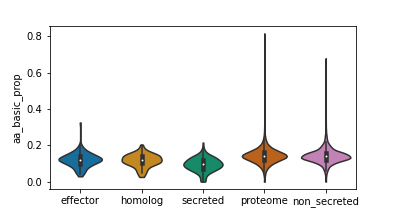


Supp. Figure 7

Proportion of basic (H, K, or R) amino acids in proteins in training datasets.


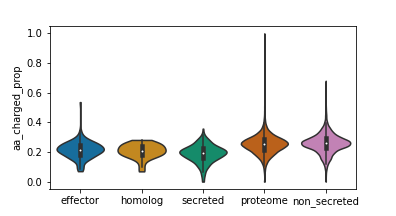


Supp. Figure 8

Proportion of charged (B, D, E, H, K, R, or Z) amino acids in proteins in training datasets.


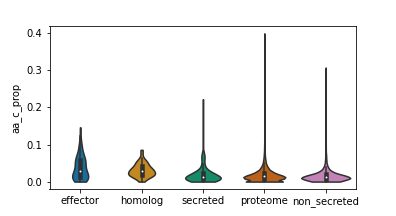


Supp. Figure 9

Proportion of cysteine amino acids in proteins in training datasets.


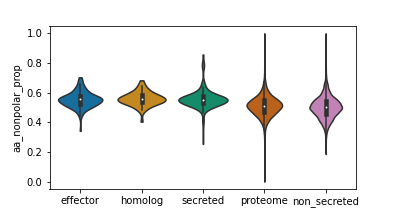


Supp. Figure 10

Proportion of non-polar (A, C, F, G, I, L, M, P, V, W, or Y) amino acids in proteins in training datasets.


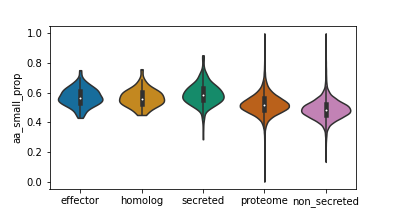


Supp. Figure 11

Proportion of small (A, B, C, D, G, N, P, S, T, or V) amino acids in proteins in training datasets.


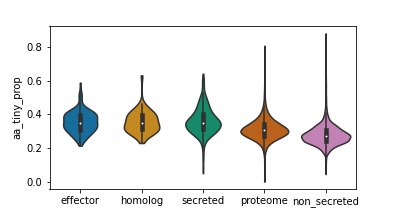


Supp. Figure 12

Proportion of small (A, C, G, S, or T) amino acids in proteins in training datasets.


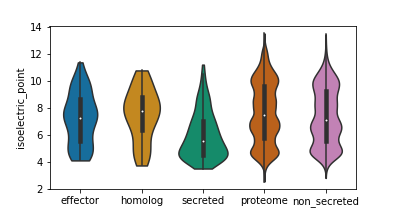


Supp. Figure 13

Distributions of protein isoelectric points in training datasets.


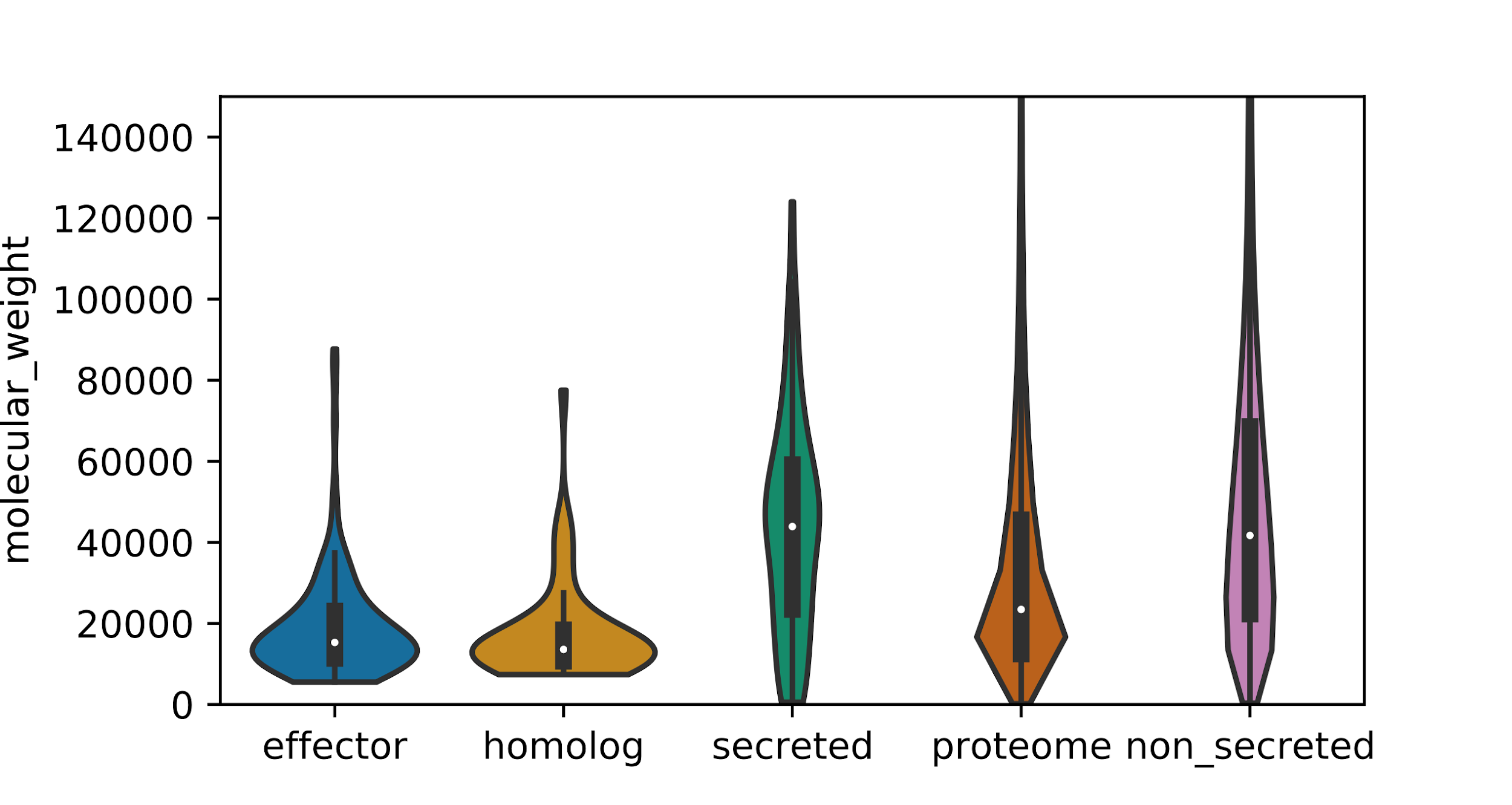


Supp. Figure 14

Distributions of molecular weight in Daltons for proteins in the training datasets.


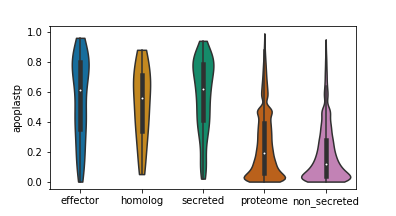


Supp. Figure 15

ApoplastP pseudo-probability of apoplastic localisation for proteins in training datasets.


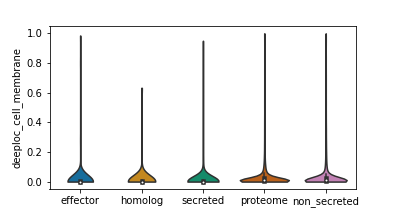


Supp. Figure 16

DeepLoc pseudo-probability of cell membrane localisation for proteins in training datasets. This probability and all other DeepLoc scores except membrane are from a multiclass classifier and sum to one for each individual protein.


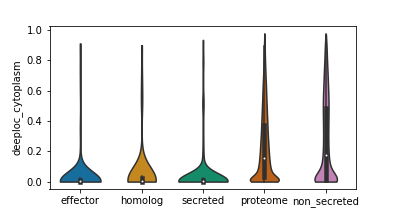


Supp. Figure 17

DeepLoc pseudo-probability of cytoplasmic localisation for proteins in training datasets. This probability and all other DeepLoc scores except membrane are from a multiclass classifier and sum to one for each individual protein.


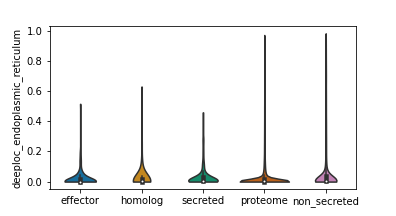


Supp. Figure 18

DeepLoc pseudo-probability of endoplasmic reticulum localisation for proteins in training datasets. This probability and all other DeepLoc scores except membrane are from a multiclass classifier and sum to one for each individual protein.


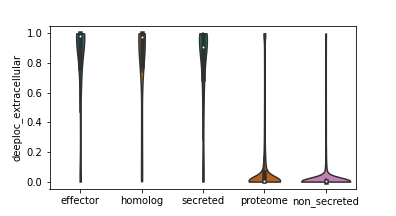


Supp. Figure 19

DeepLoc pseudo-probability of extracellular localisation for proteins in training datasets. This probability and all other DeepLoc scores except membrane are from a multiclass classifier and sum to one for each individual protein.


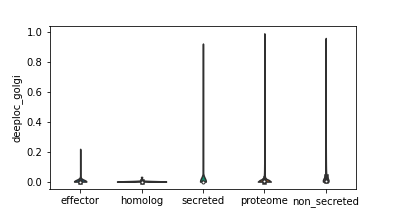


Supp. Figure 20

DeepLoc pseudo-probability of golgi localisation for proteins in training datasets. This probability and all other DeepLoc scores except membrane are from a multiclass classifier and sum to one for each individual protein.


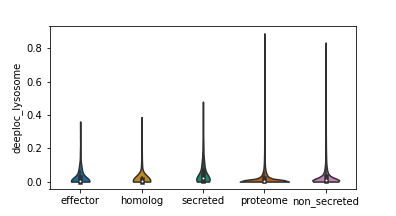


Supp. Figure 21

DeepLoc pseudo-probability of lysosome localisation for proteins in training datasets. This probability and all other DeepLoc scores except membrane are from a multiclass classifier and sum to one for each individual protein.


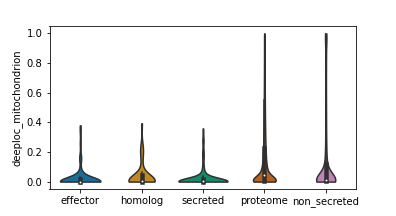


Supp. Figure 22

DeepLoc pseudo-probability of mitochondrial localisation for proteins in training datasets. This probability and all other DeepLoc scores except membrane are from a multiclass classifier and sum to one for each individual protein.


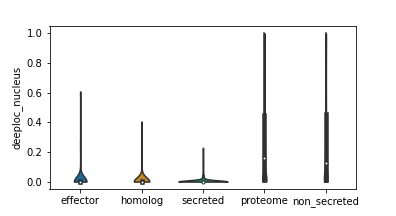


Supp. Figure 23

DeepLoc pseudo-probability of nuclear localisation for proteins in training datasets. This probability and all other DeepLoc scores except membrane are from a multiclass classifier and sum to one for each individual protein.


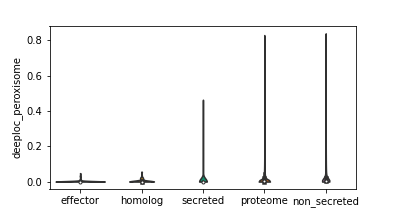


Supp. Figure 24

DeepLoc pseudo-probability of peroxisomal localisation for proteins in training datasets. This probability and all other DeepLoc scores except membrane are from a multiclass classifier and sum to one for each individual protein.


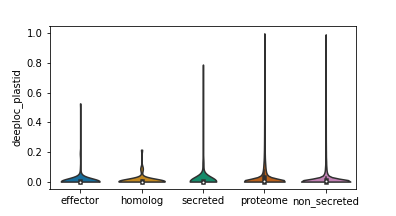


Supp. Figure 25

DeepLoc pseudo-probability of plastid localisation for proteins in training datasets. This probability and all other DeepLoc scores except membrane are from a multiclass classifier and sum to one for each individual protein.


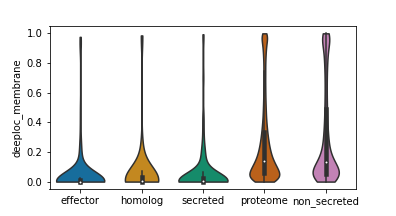


Supp. Figure 26

DeepLoc pseudo-probability of membrane association for proteins in training datasets.


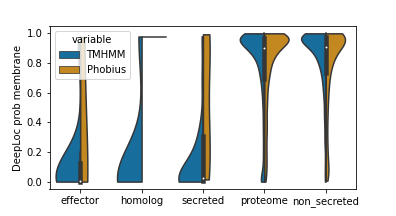


Supp. Figure 27

Split violin plot showing DeepLoc pseudo-probability of membrane association for proteins in the training dataset with at least one transmembrane domain predicted by TMHMM (left) or Phobius (right).


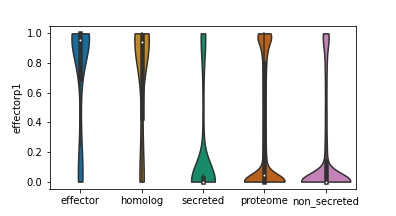


Supp. Figure 28

EffectorP 1 pseudo-probability of effector classification for proteins in the training dataset. This distribution is on the full dataset, including proteins without signal peptides.


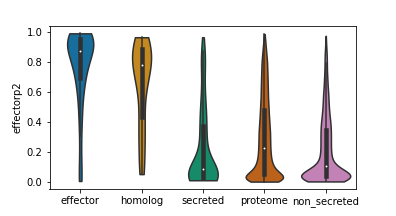


Supp. Figure 29

EffectorP 2 pseudo-probability of effector classification for proteins in the training dataset. This distribution is on the full dataset, including proteins without signal peptides.


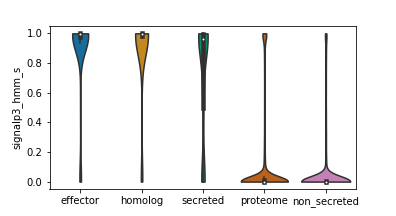


Supp. Figure 30

Distributions of SignalP 3 - HMM S-scores for proteins in the training dataset.


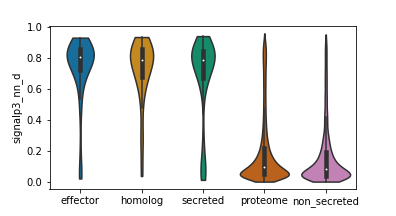


Supp. Figure 31

Distributions of SignalP 3 - NN D-scores for proteins in the training dataset.


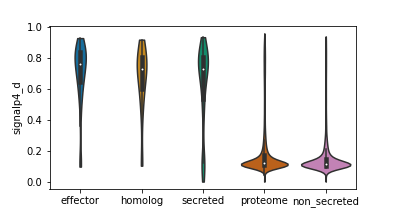


Supp. Figure 32

Distributions of SignalP 4 D-scores for proteins in the training dataset.


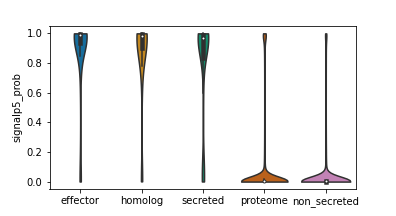


Supp. Figure 33

Distributions of SignalP 5 pseudo-probabilities of having a signal peptide for proteins in the training dataset.


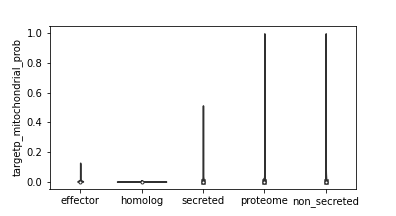


Supp. Figure 34

Distributions of TargetP pseudo-probabilities of having a mitochondrial target peptide for proteins in the training dataset. This probability and all other TargetP scores are from a multiclass classifier and sum to one for each individual protein.


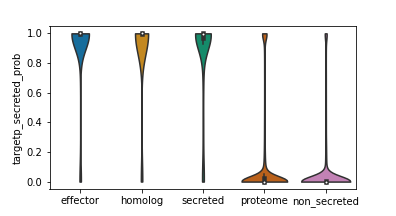


Supp. Figure 35

Distributions of TargetP pseudo-probabilities of having a secretion target peptide for proteins in the training dataset. This probability and all other TargetP scores are from a multiclass classifier and sum to one for each individual protein.


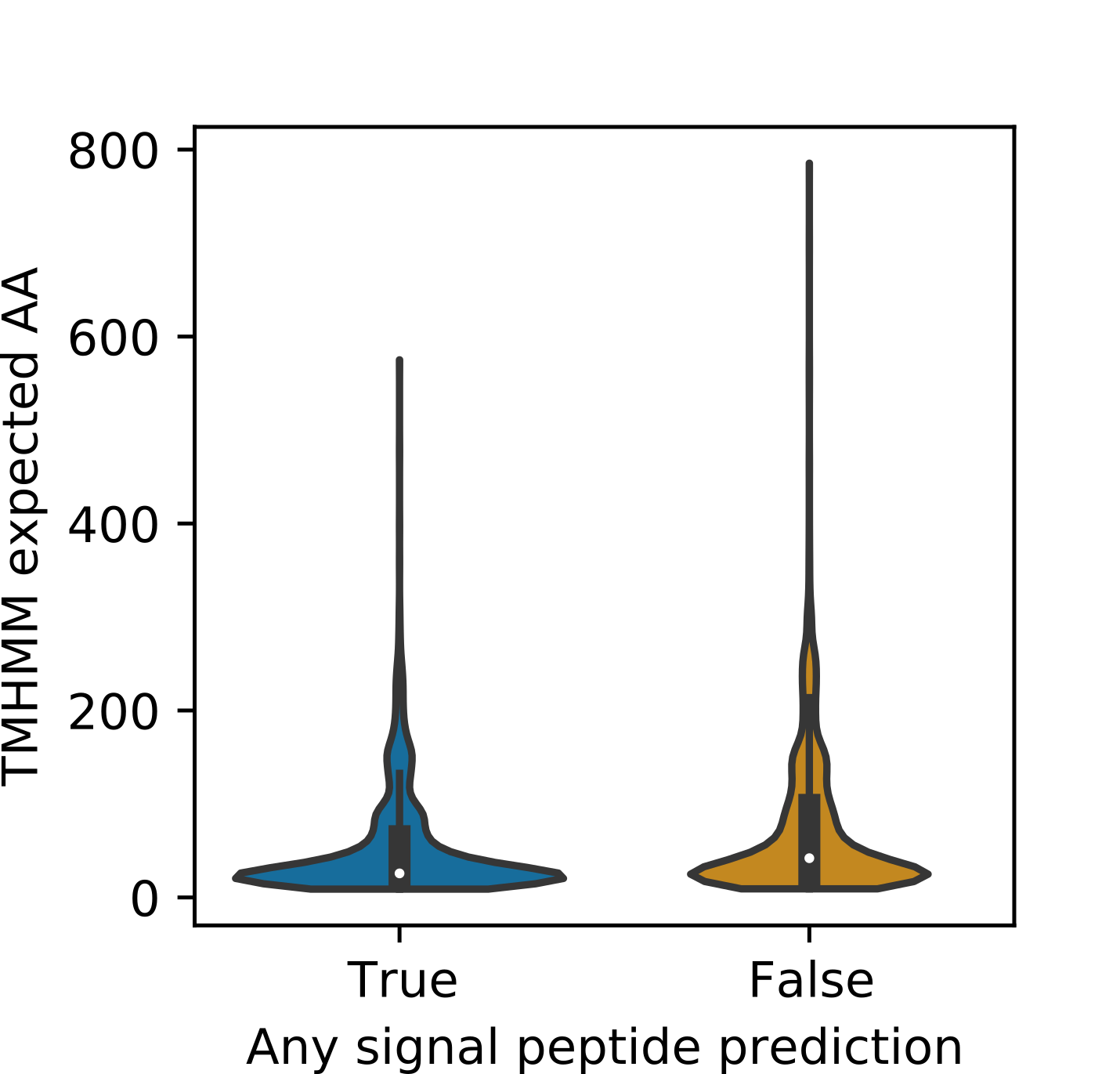


Supp. Figure 36

Distributions of TMHMM expected number of AAs in transmembrane domains for proteins with and without a signal peptide, for proteins in the training dataset.


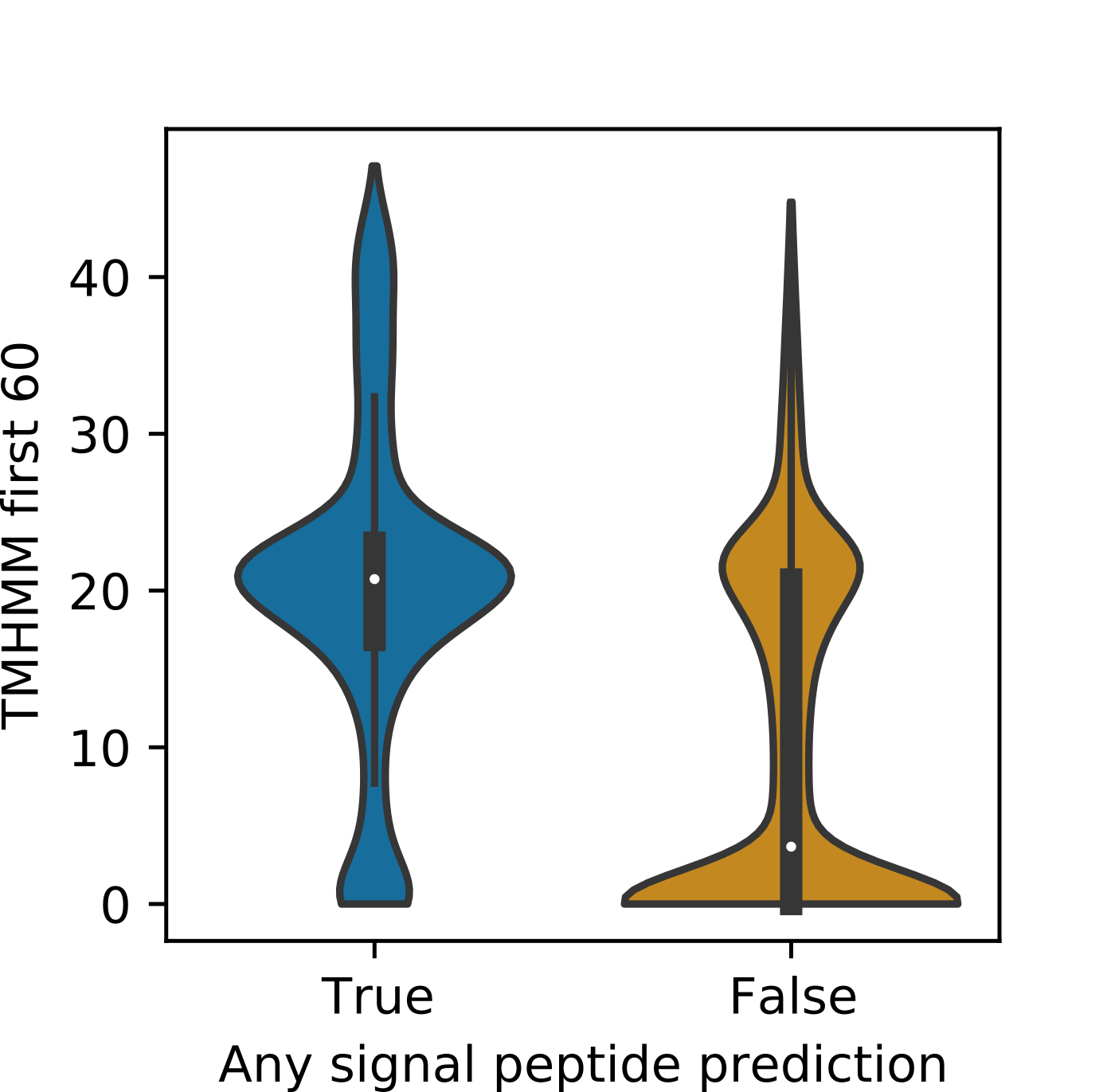


Supp. Figure 37

Distributions of TMHMM expected number of AAs in transmembrane domains in the first 60 residues for proteins with and without a signal peptide, for proteins in the training dataset.


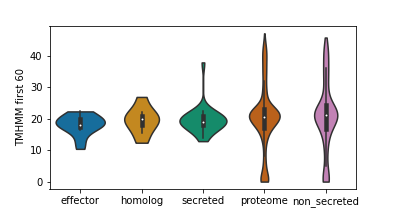


Supp. Figure 38

Distributions of TMHMM expected number of AAs in transmembrane domains in the first 60 residues for proteins in the training datasets.


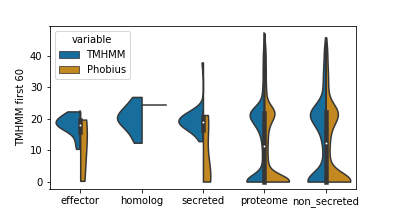


Supp. Figure 39

Split violin plot showing the distribution TMHMM expected number of AAs in transmembrane domains in the first 60 residues for proteins for proteins predicted to have at least one transmembrane domain by TMHMM (left) of Phobius (right), for each class in the training dataset.


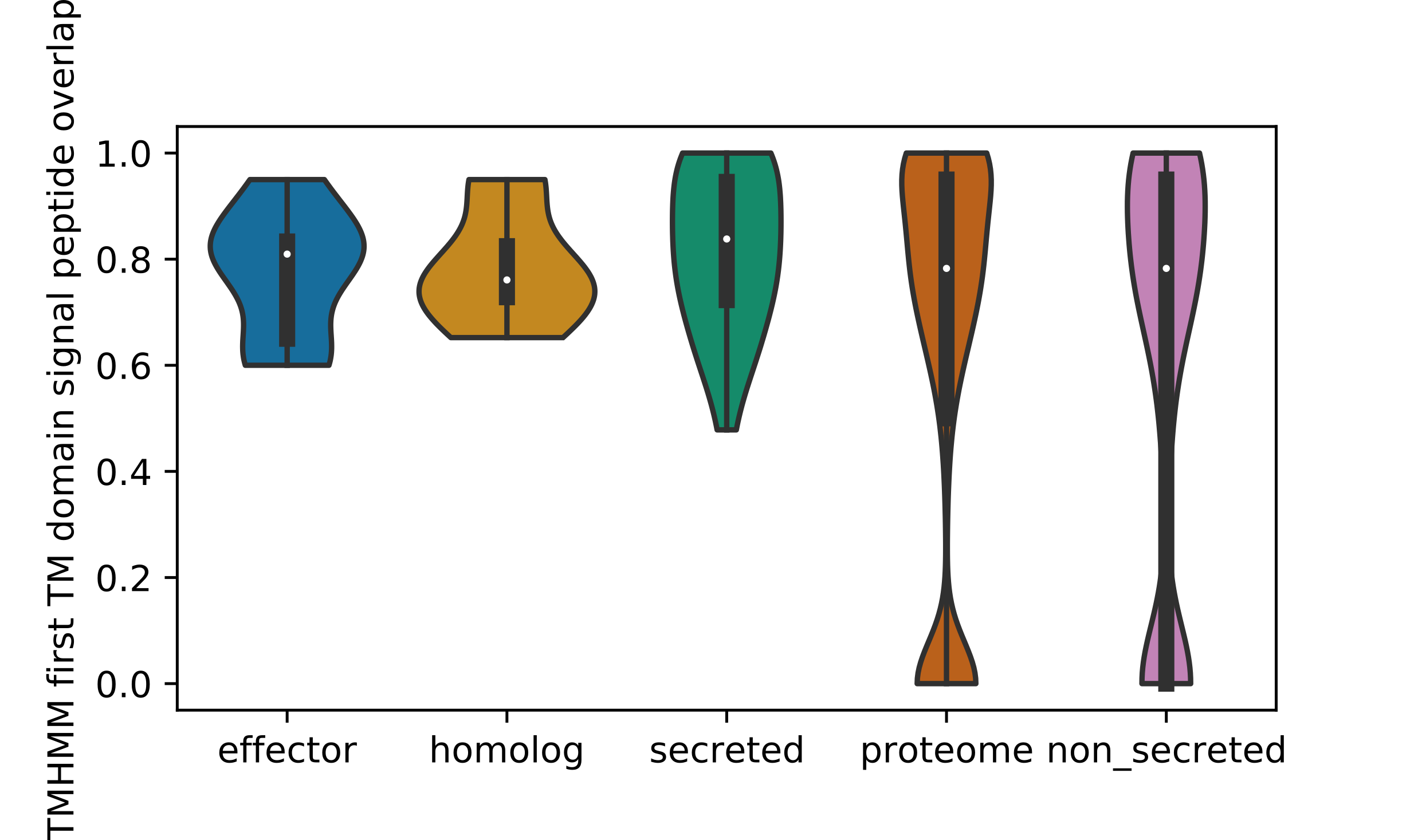


Supp. Figure 40

Distribution of proportion of overlap between the median predicted signal peptide cut-site and the first transmembrane domain predicted by TMHMM. Only includes proteins with both a signal peptide and at least one transmembrane domain in the training datasets.


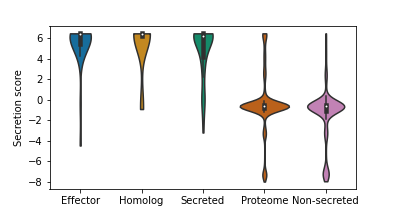


Supp. Figure 41

Manually created secretion score with default weights, for each class in the test dataset.


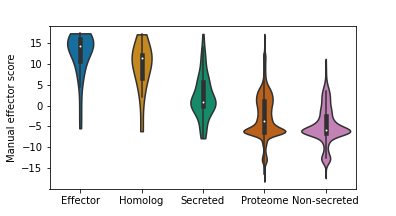


Supp. Figure 42

Manually created effector score with default weights for each class in the test dataset.


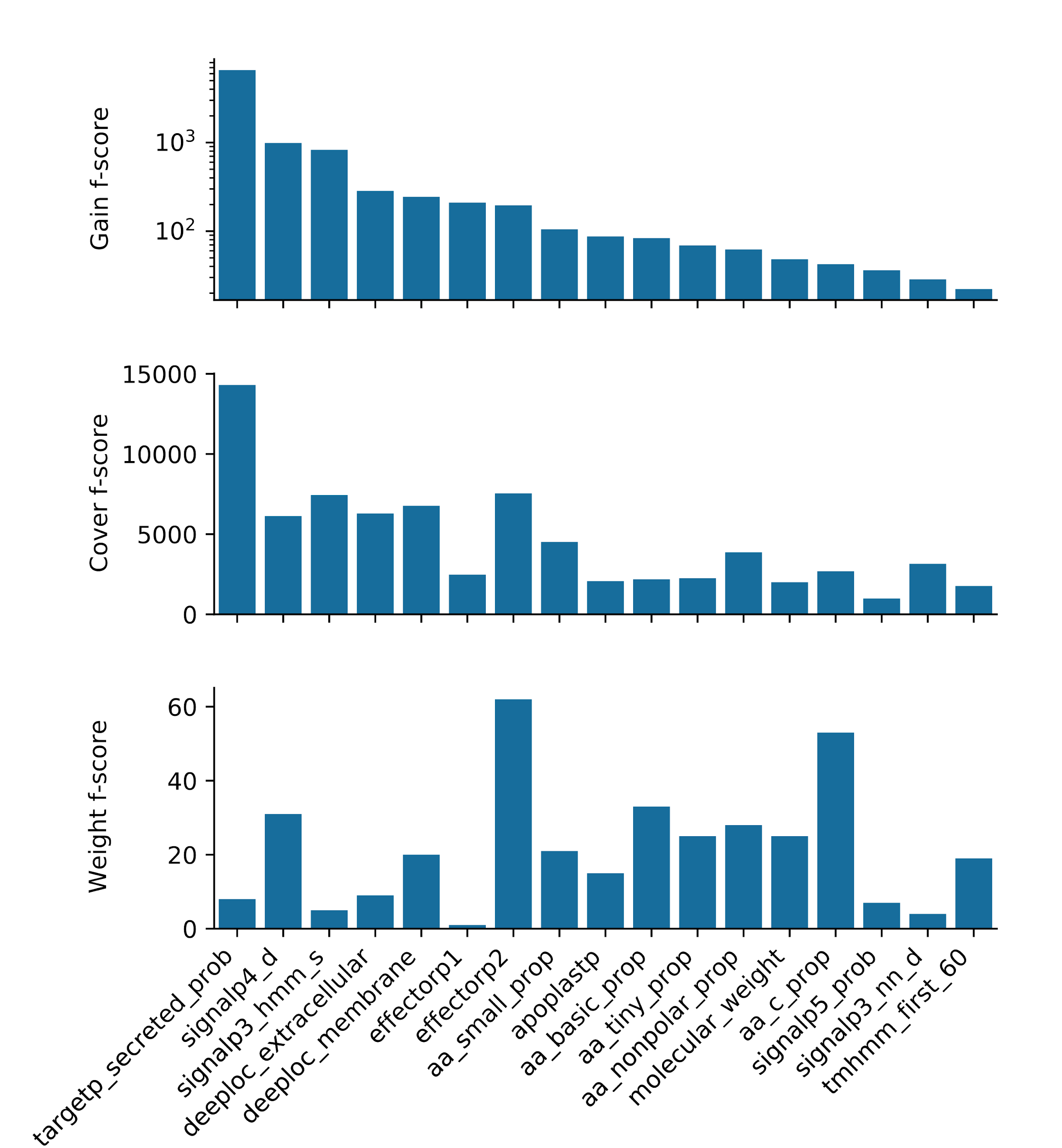


Supp. Figure 43

Feature importances for the trained learning-to-rank (LTR) model.

Gain is the improvement in score given by branches that use the feature and is the best indicator of feature importance generally (note the log scale). Cover is the average number of samples that are affected by each split. Weight is how many times a feature is used in the trees.


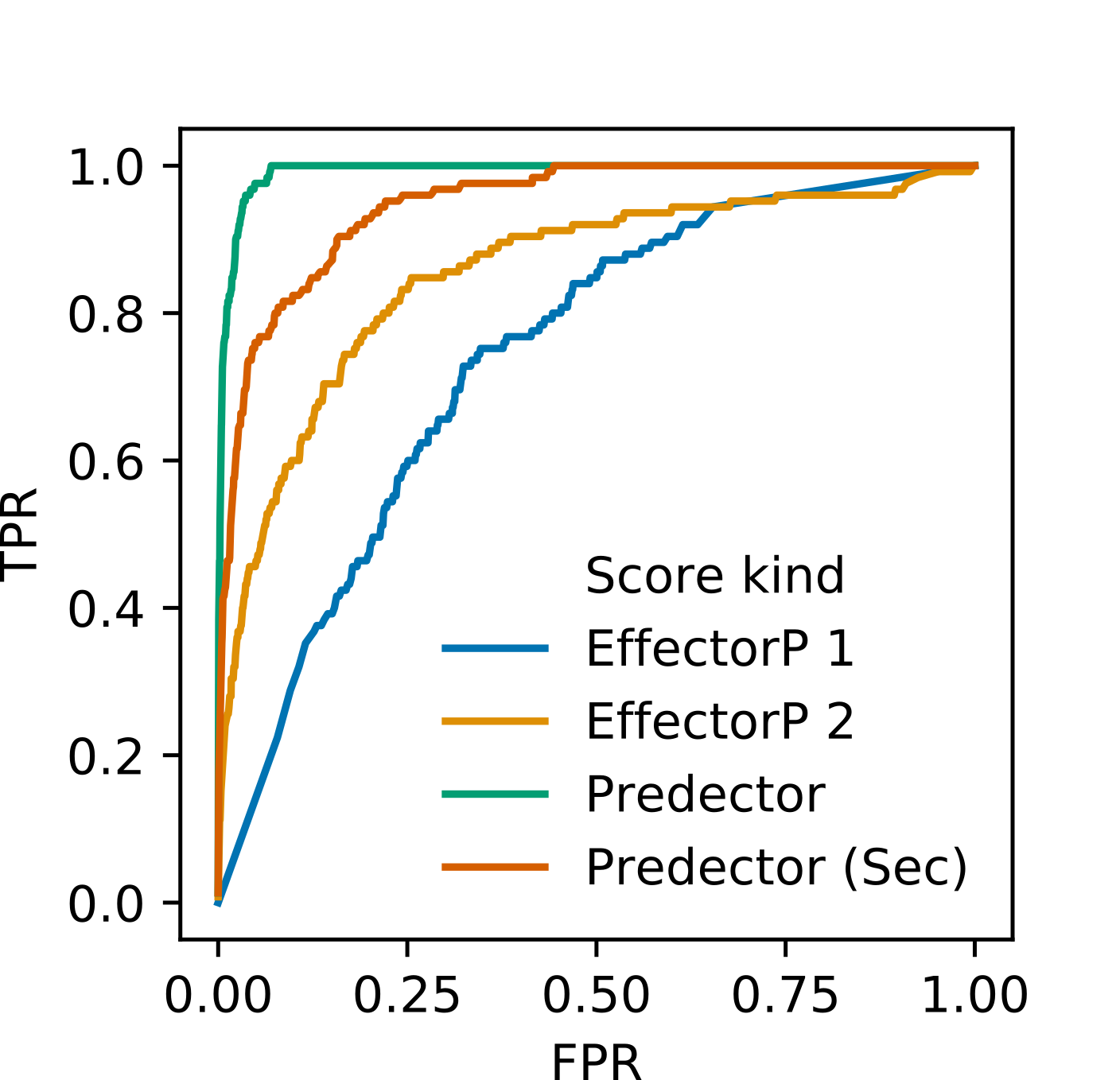


Supp. Figure 44

Receiver operator characteristic (ROC) curves for the training dataset for Predector and EffectorP scores. EffectorP 1 and 2, and “Predector (Sec)” were only tested on the subset of proteins predicted to be secreted. The curve for “Predector” is evaluated on the full dataset.


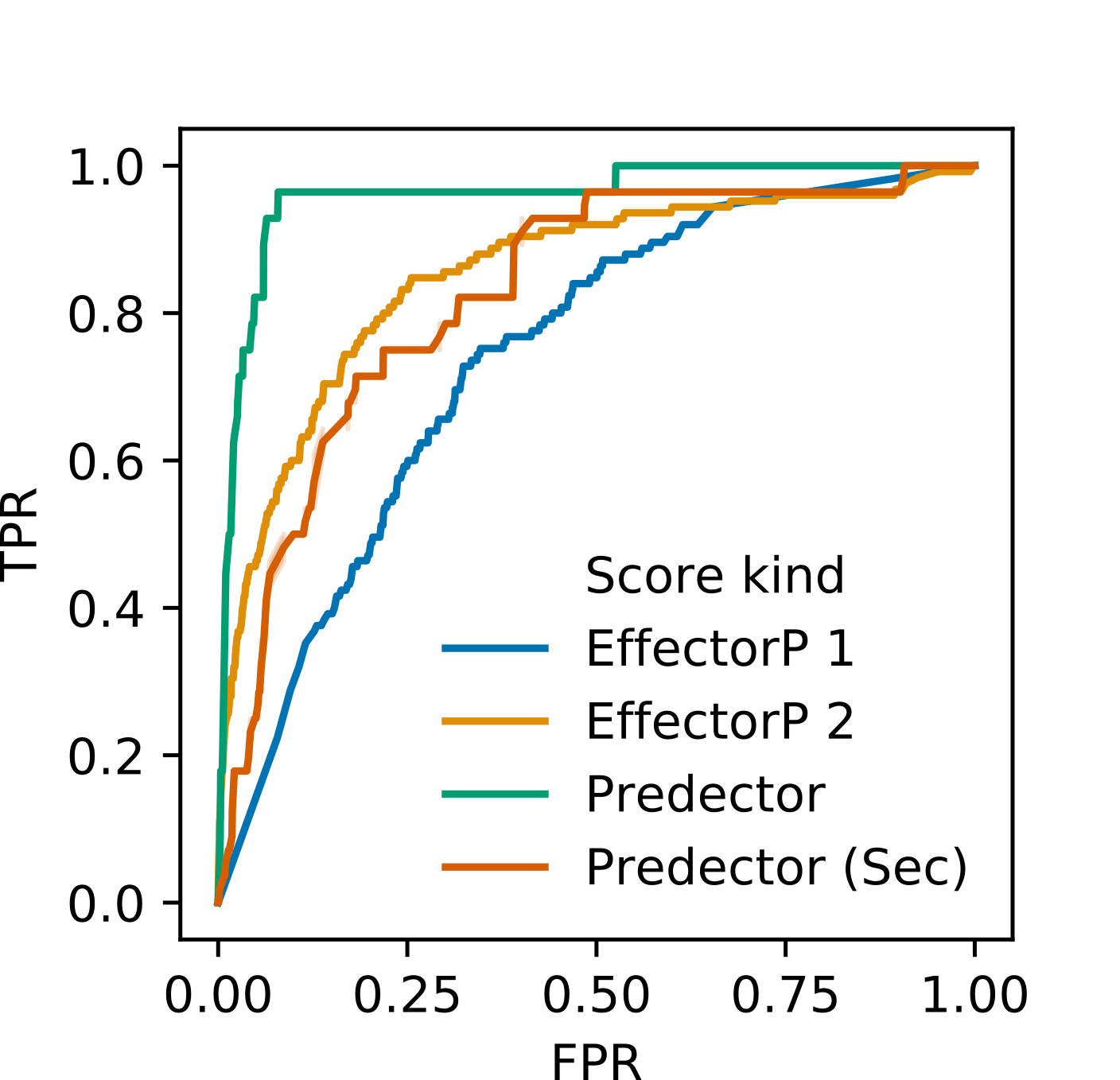


Supp. Figure 45

Receiver operator characteristic (ROC) curves for the testing dataset for Predector and EffectorP scores. EffectorP 1 and 2, and “Predector (Sec)” were only tested on the subset of proteins predicted to be secreted. The curve for “Predector” is evaluated on the full dataset.


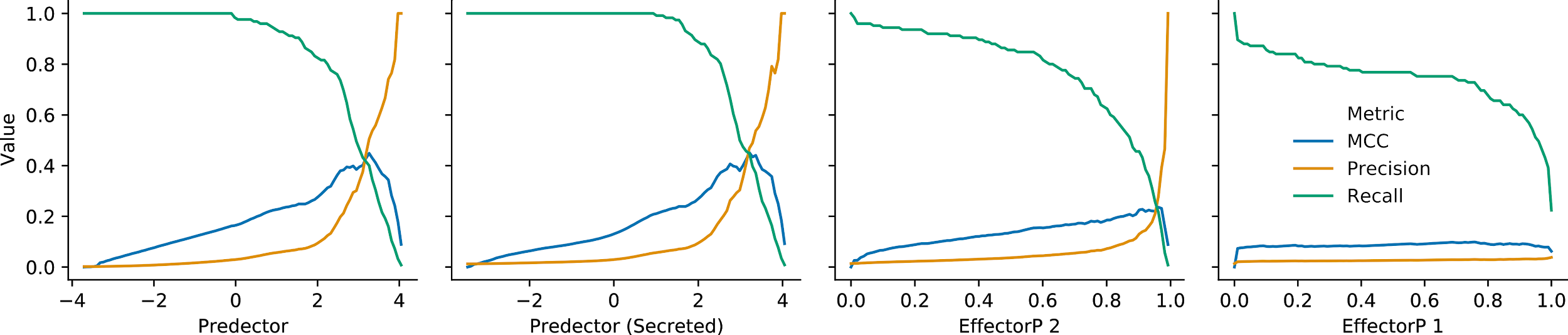


Supp. Figure 46

Precision, Recall, and MCC at 100 different binary classification thresholds in the training data. EffectorP 1 and 2, and “Predector (Sec)” were only tested on the subset of proteins predicted to be secreted. The curves for “Predector” are evaluated on the full dataset.


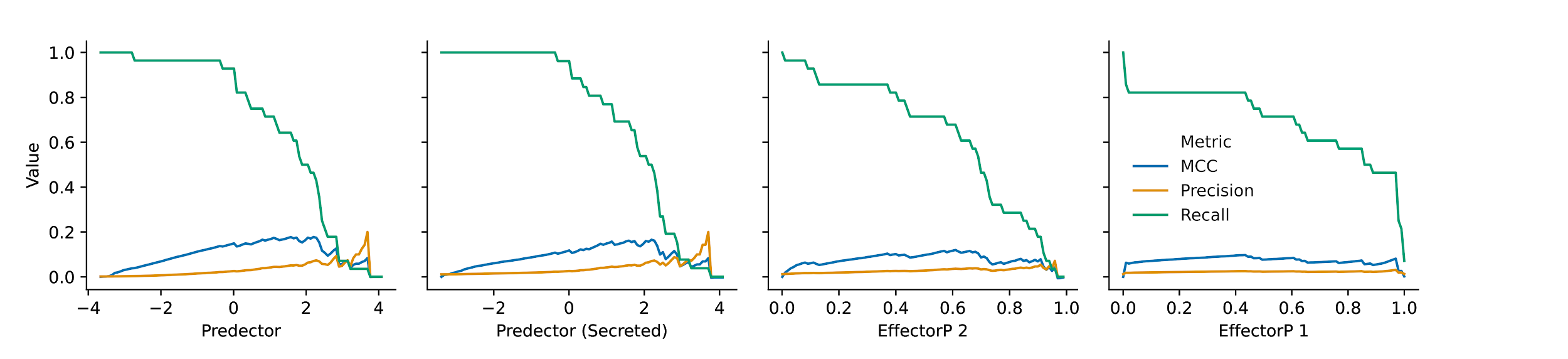


Supp. Figure 47

Precision, Recall, and MCC at 100 different binary classification thresholds in the testing data. EffectorP 1 and 2, and “Predector (Sec)” were only tested on the subset of proteins predicted to be secreted. The curves for “Predector” are evaluated on the full dataset.


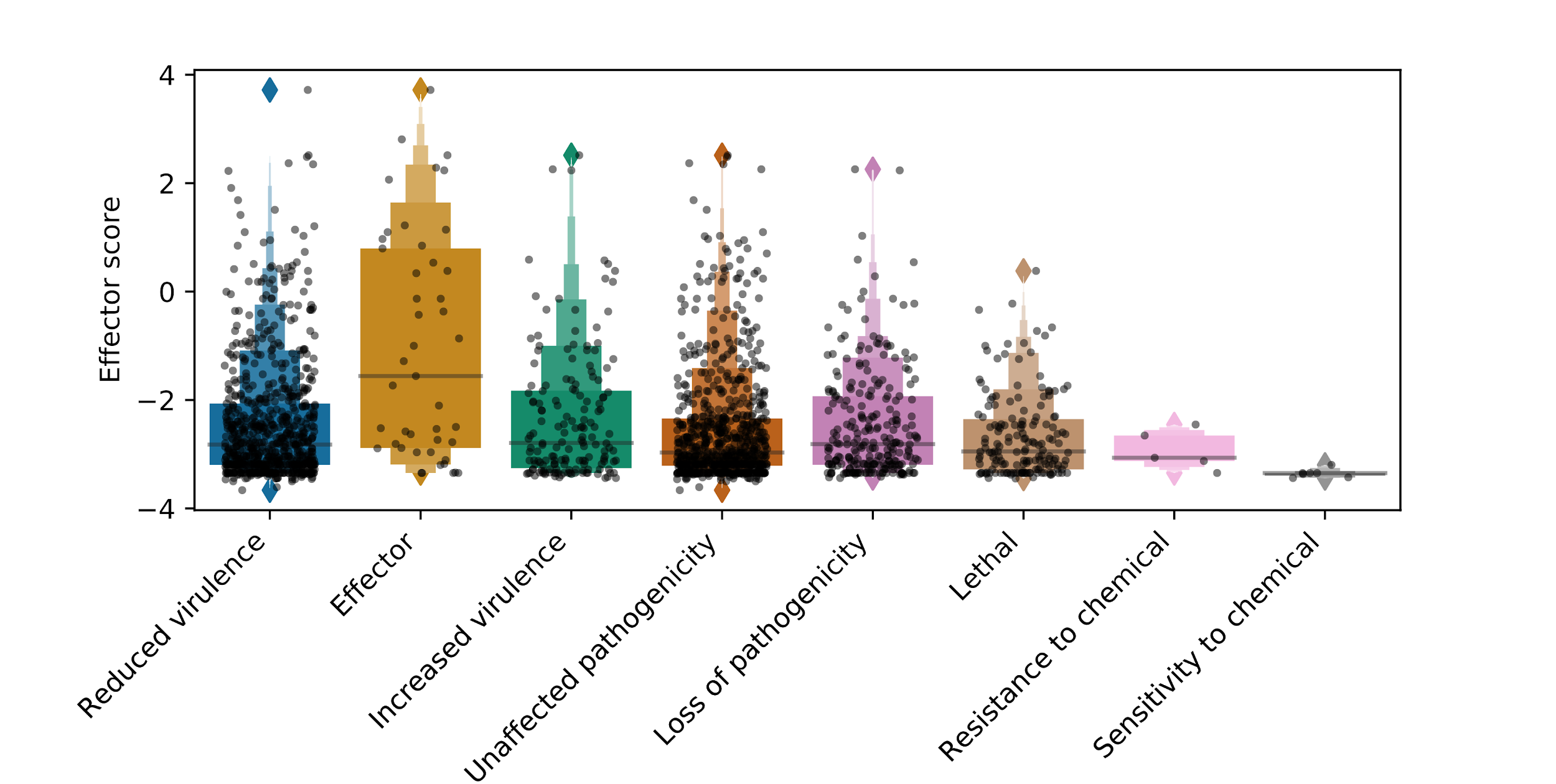


Supp. Figure 48

Distribution of Predector “effector” scores for proteins with a significant PHI-base match with each phenotype for proteins in the test dataset. Proteins may have multiple phenotypes associated with them. The effector class includes non-fungal effectors (e.g. from Bacteria or Oomycetes).


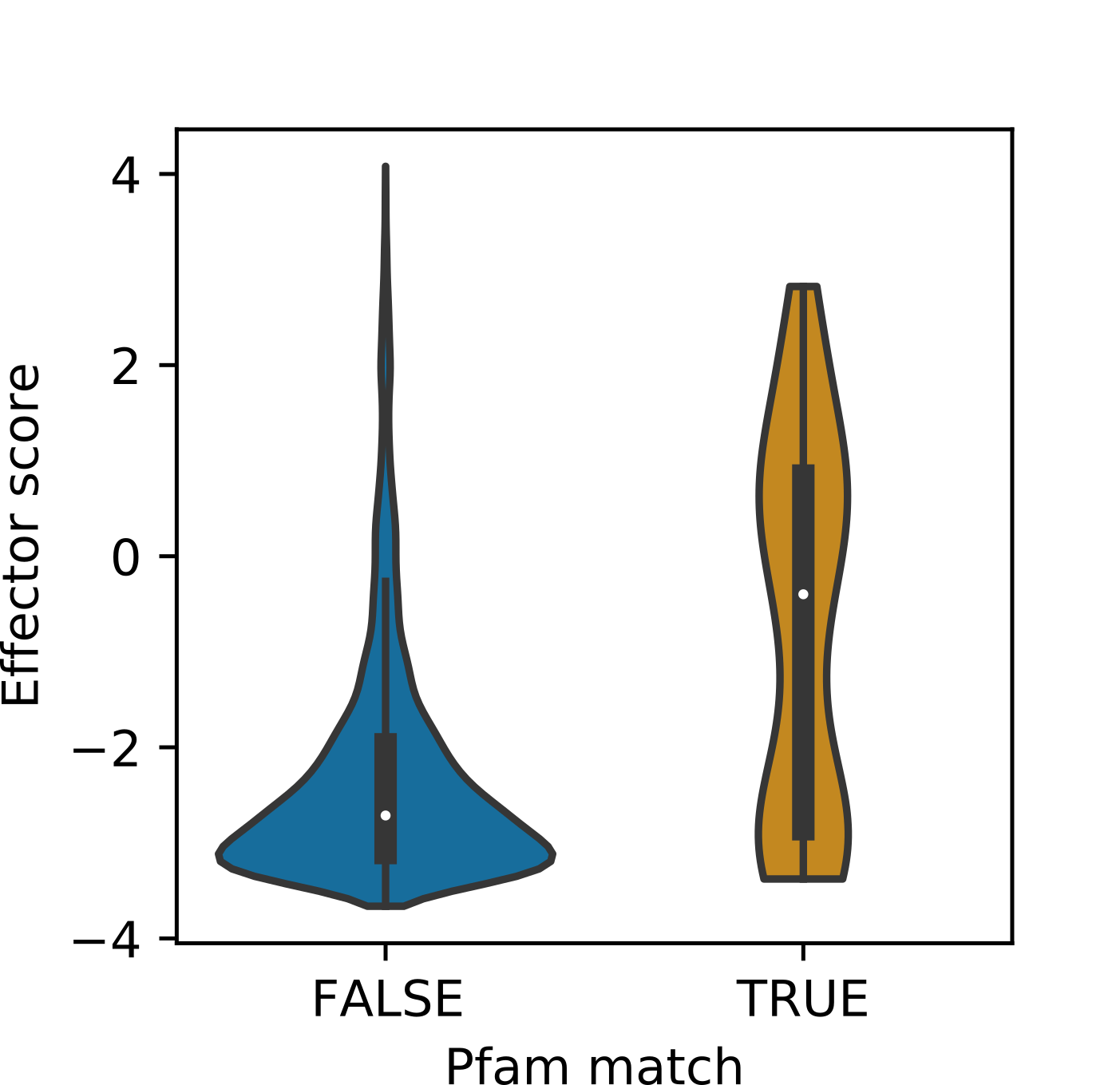


Supp. Figure 49

Distribution of Predector “effector” score for proteins with a significant match to one of the Pfam domains indicative of effector activity.


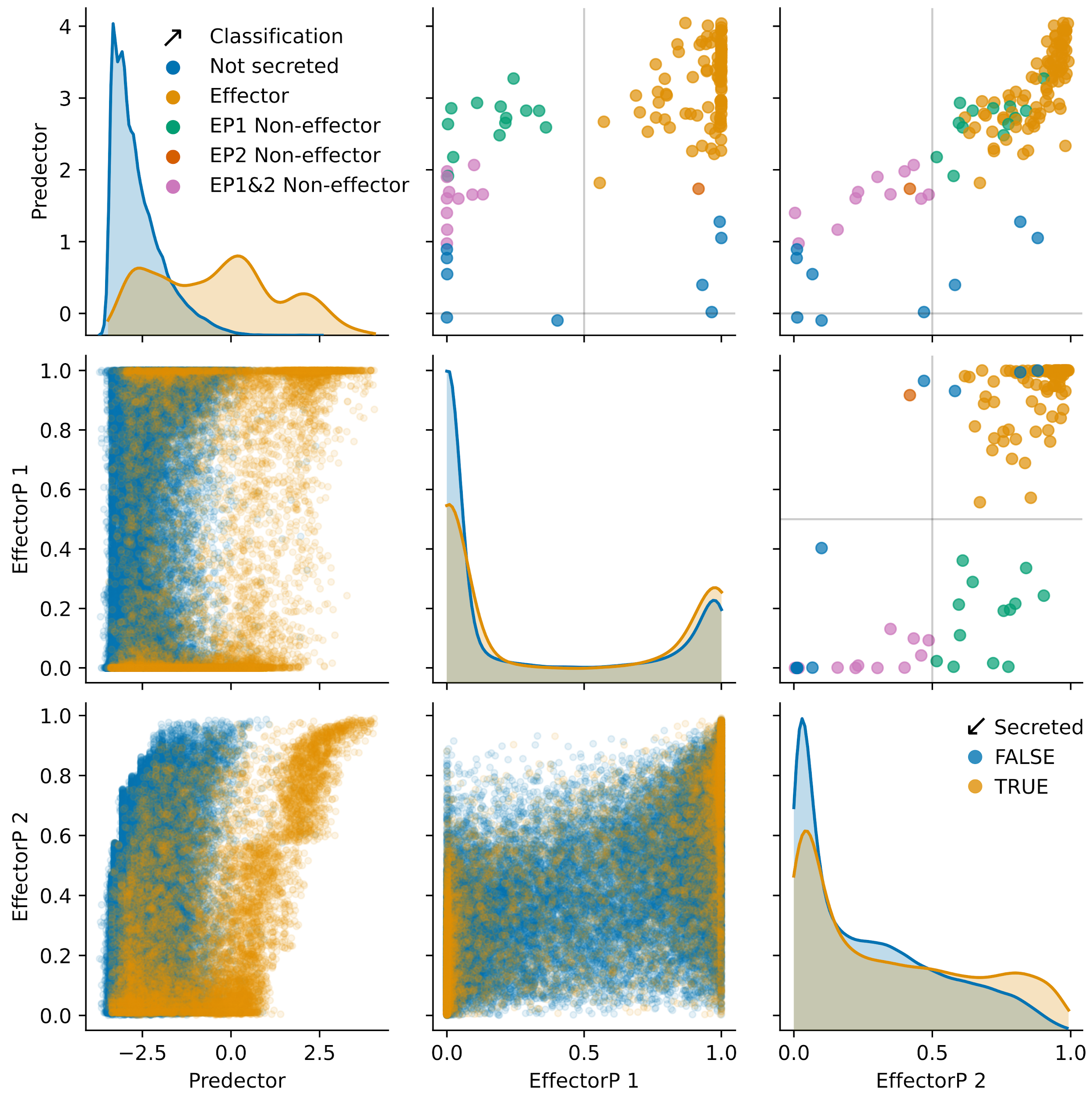


Supp. Figure 50

Comparing the scores of Predector with EffectorP versions 1 and 2 for proteins in the training dataset. Scatter plots in the lower-left corner indicate comparisons of predictive scores between methods, with predicted secreted proteins (any signal peptide and fewer than two TM domains predicted) indicated in yellow, and non-secreted proteins indicated in blue. Density plots along the diagonal indicate distributions of the full test dataset versus predictive scores for each method (indicated along the x-axis), also coloured by secretion prediction as before (Note: there are far more non-secreted than secreted proteins in the dataset). Scatter plots in the top-right corner indicate score comparisons between methods for confirmed effectors, coloured by whether they have been predicted as secreted (criteria as above), or additionally predicted by EffectorP versions 1 or 2.


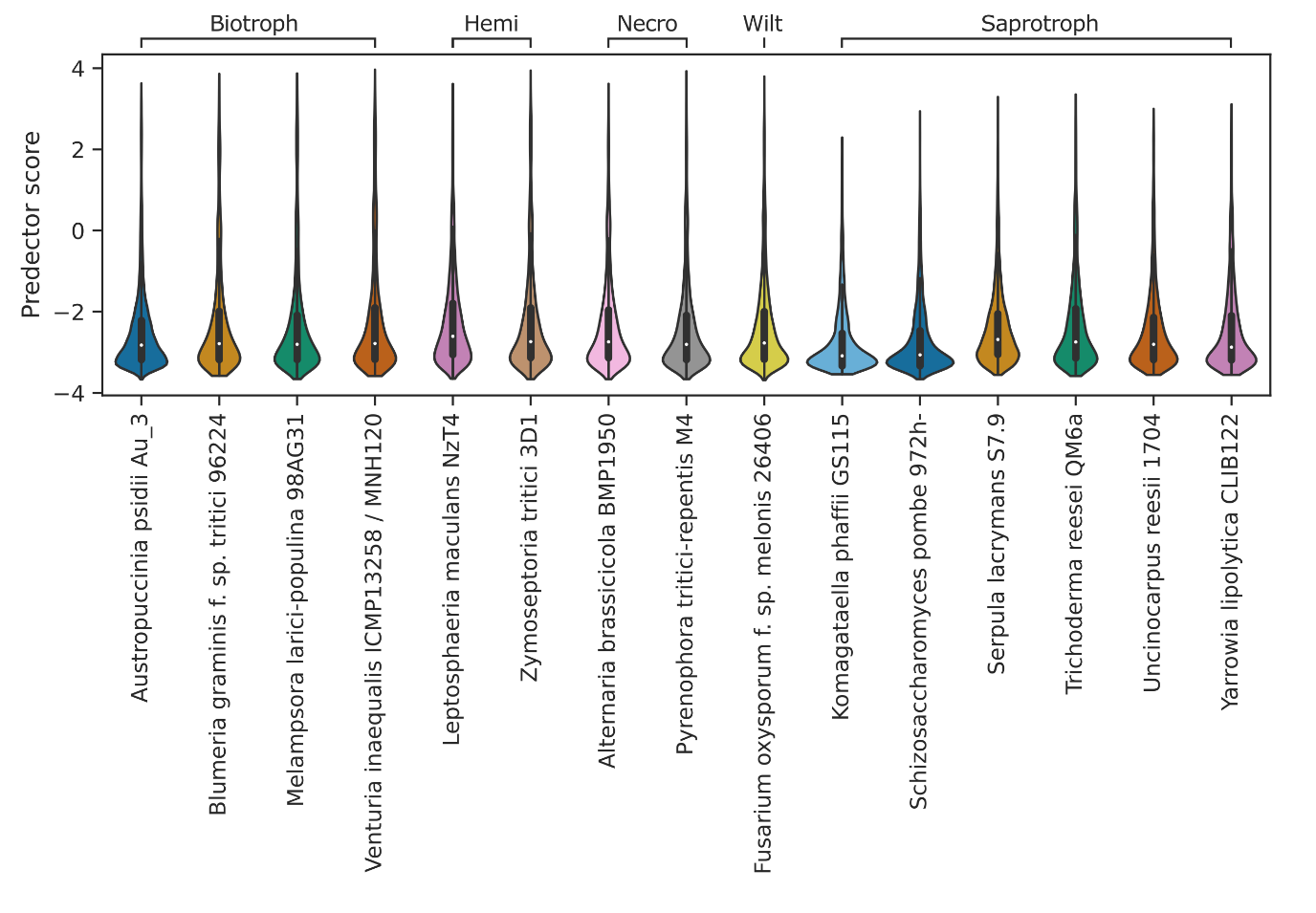


Supp. Figure 51.

Predector score distribution for multiple fungal proteomes. On the left hand side (Austropuccinia to Fusarium) are a number of common pathogens, and on the right (Komagataella to Yarrowia) are a number of saprotrophs.


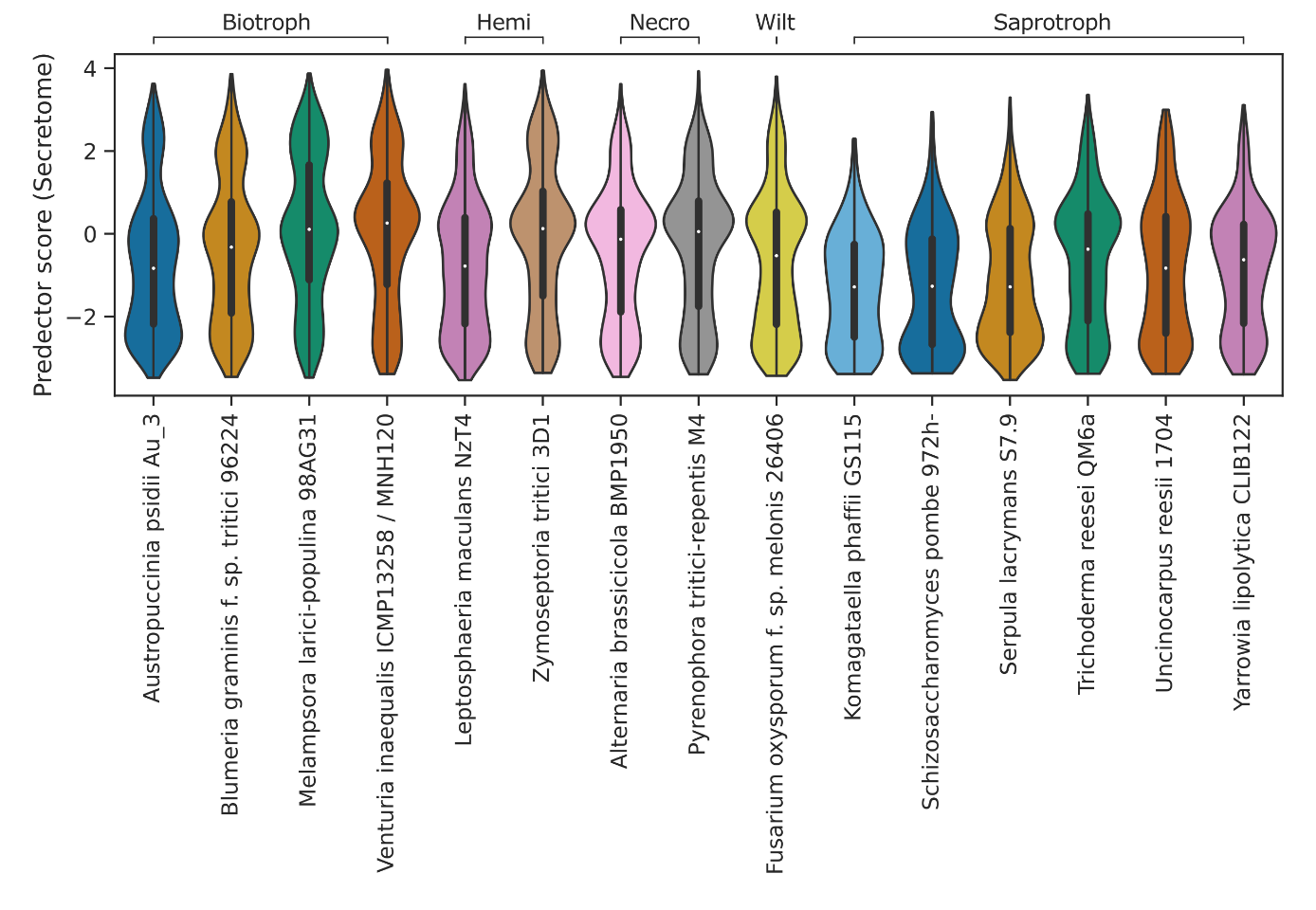


Supp. Figure 52.

Predector score distribution for multiple fungal proteomes restricted to the predicted secretomes (defined as having a signal peptide prediction by any method, and fewer than 2 transmembrane domain predictions). On the left hand side (Austropuccinia to Fusarium) are a number of common pathogens, and on the right (Komagataella to Yarrowia) are a number of saprotrophs.


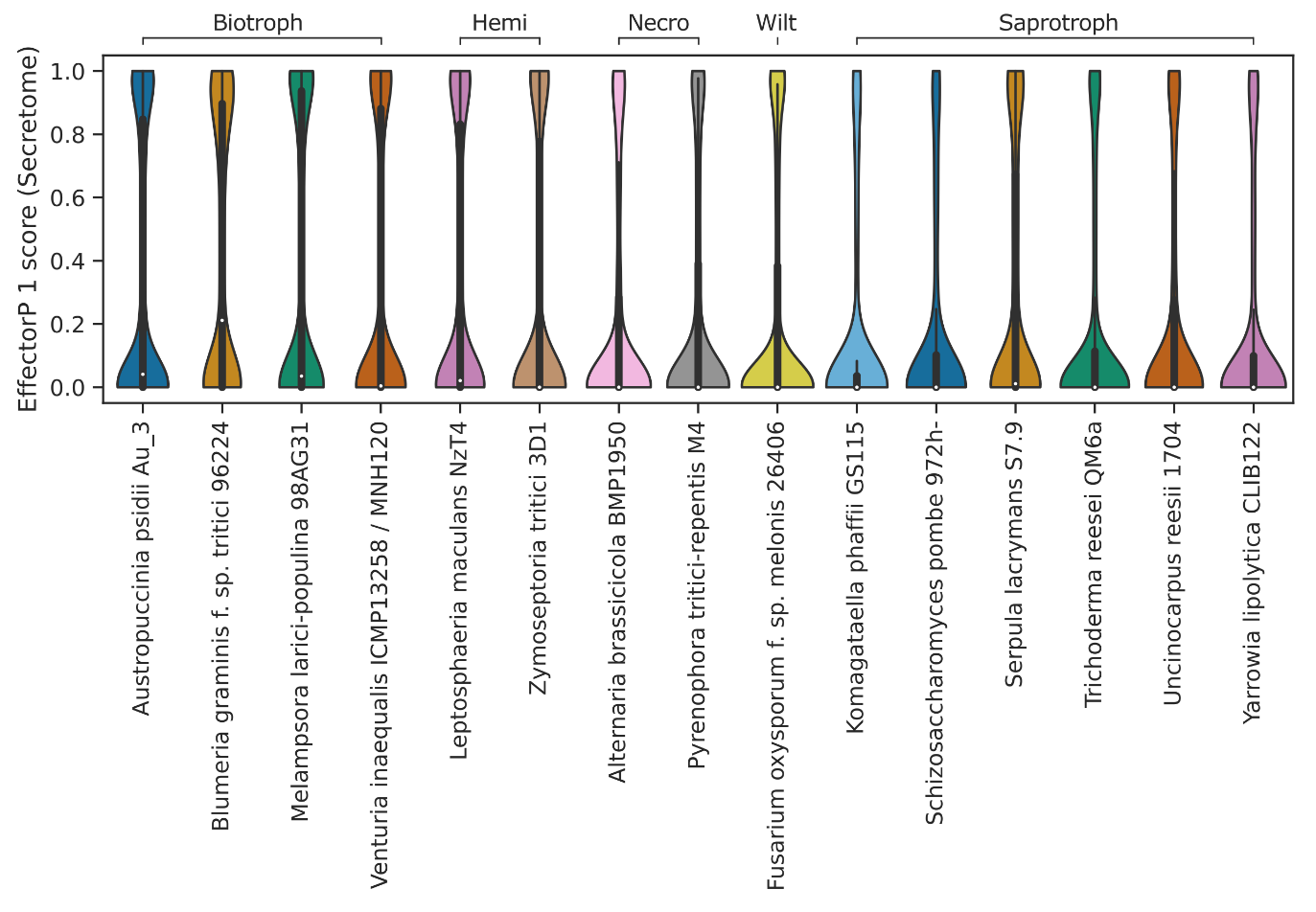


Supp. Figure 53.

EffectorP1 score distribution for multiple fungal proteomes restricted to the predicted secretomes (defined as having a signal peptide prediction by any method, and fewer than 2 transmembrane domain predictions). On the left hand side (Austropuccinia to Fusarium) are a number of common pathogens, and on the right (Komagataella to Yarrowia) are a number of saprotrophs.


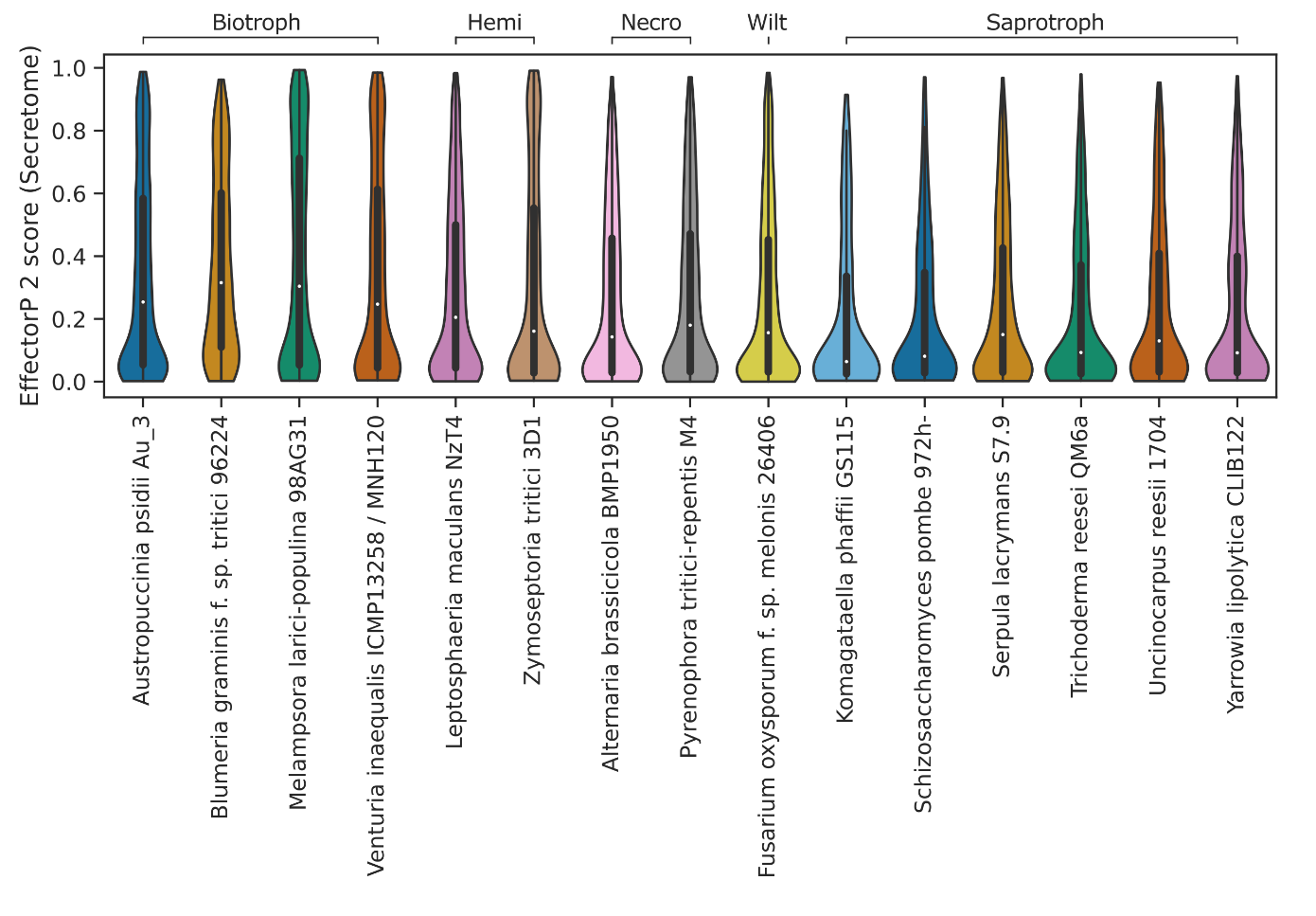


Supp. Figure 54.

EffectorP2 score distribution for multiple fungal proteomes restricted to the predicted secretomes (defined as having a signal peptide prediction by any method, and fewer than 2 transmembrane domain predictions). On the left hand side (Austropuccinia to Fusarium) are a number of common pathogens, and on the right (Komagataella to Yarrowia) are a number of saprotrophs.
